# Tumor Immunotherapy and Microbiome: From Bench‐to‐Bedside Applications

**DOI:** 10.1002/mco2.70454

**Published:** 2026-01-20

**Authors:** Anqi Lin, Minying Xiong, Aimin Jiang, Li Chen, Lihaoyun Huang, Kailai Li, Hank Z. H. Wong, Jian Zhang, Zaoqu Liu, Quan Cheng, Bufu Tang, Pengpeng Zhang, Peng Luo

**Affiliations:** ^1^ Department of Oncology Zhujiang Hospital, Southern Medical University Guangzhou China; ^2^ Department of Urology Changhai Hospital, Naval Medical University (Second Military Medical University) Shanghai China; ^3^ Cancer Centre and Institute of Translational Medicine, Faculty of Health Sciences University of Macau Macau SAR China; ^4^ Li Ka Shing Faculty of Medicine The University of Hong Kong Hong Kong SAR China; ^5^ Institute of Basic Medical Sciences Chinese Academy of Medical Sciences and Peking Union Medical College Beijing China; ^6^ Department of Neurosurgery Xiangya Hospital, Central South University Changsha China; ^7^ National Clinical Research Center for Geriatric Disorders Xiangya Hospital, Central South University Changsha China; ^8^ Department of Interventional Radiology Zhongshan Hospital, Fudan University Shanghai China; ^9^ Department of Lung Cancer Tianjin Lung Cancer Center National Clinical Research Center for Cancer Key Laboratory of Cancer Prevention and Therapy Tianjin's Clinical Research Center for Cancer Tianjin Medical University Cancer Institute and Hospital Tianjin China; ^10^ School of Clinical Medicine, Li Ka Shing Faculty of Medicine The University of Hong Kong Hong Kong SAR China

**Keywords:** cancer, immune checkpoint inhibitors, immunotherapy, microbiome, tumor microenvironment

## Abstract

Cancer immunotherapy has emerged as a transformative therapeutic strategy that harnesses the immune system to combat malignant tumors, overcoming critical limitations such as the nonspecific cytotoxicity of conventional chemotherapy and radiotherapy and drug resistance arising from target mutations in targeted therapies. Growing evidence demonstrates that the human microbiome plays a pivotal role in modulating immune responses and influencing the efficacy of immunotherapeutic interventions. Although the impact is increasingly recognized, the molecular mechanisms and translational potential of microbiome‐based strategies remain incompletely explored. This review systematically elucidates how microorganisms from distinct anatomical sites (including bacteria, fungi, and viruses residing in the gut, oral cavity, skin, respiratory tract, and urogenital tract) and intratumoral microbes modulate the tumor immune microenvironment through metabolites, immune cell priming, and antigen mimicry. Furthermore, we discuss how specific microbial signatures predict responses to immune checkpoint inhibitors (ICIs) and CAR‐T cell therapy, and highlight emerging interventional strategies, including fecal microbiome transplantation (FMT), probiotics, and engineered bacteria, that demonstrate synergistic effects with immunotherapy in preclinical and clinical settings. By integrating mechanistic insights with translational advances, this review provides a comprehensive scientific foundation for microbiome‐based precision immunotherapy, aimed at improving patient survival outcomes and reducing treatment‐related adverse events.

## Graphical abstract

The microbiome is related to the efficacy of immunotherapy and can be utilized to predict the efficacy and adverse reactions of immunotherapy. Microbiome‐targeted intervention strategies can improve the efficacy of ICI, but necessitating more comprehensive exploration.

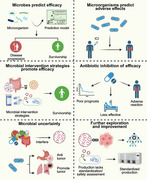

## 1 Introduction

Cancer immunotherapy is a therapeutic strategy that combats cancer by strategically modulating immune system function [1, 2, 3, 4, 5, 6, 7]. In 1893, American orthopedic surgeon William Coley injected bacteria into tumor tissues, documenting the first significant tumor regression, thus marking the historical inception of cancer immunotherapy [8]. Subsequently, scientists discovered key immune checkpoint molecules involved in tumor immune evasion, including cytotoxic T‐lymphocyte‐associated antigen 4 (CTLA‐4) and programmed cell death protein 1 (PD‐1), which led to the development of a series of immune checkpoint inhibitors (ICIs) [9, 10, 11, 12, 13, 14]. This discovery facilitated the translation of fundamental scientific research into innovative clinical therapeutic strategies, thereby advancing the development of cancer immunotherapy. From a molecular mechanism perspective, cancer cells can establish an immune‐evasive microenvironment through various pathways, including the production of immunosuppressive factors, upregulation of immune checkpoint proteins, and downregulation of major histocompatibility complex (MHC) molecules, thereby effectively evading the immune system's surveillance and cytotoxic capabilities [15, 16, 17, 18, 19]. Correspondingly, cancer immunotherapy functions by counteracting these immunosuppressive mechanisms, thereby reinstating the immune system's capacity to eliminate cancer cells. Currently approved cancer immunotherapies primarily encompass ICI therapies targeting the tumor immune microenvironment, adoptive cellular immunotherapy, oncolytic virus (OV) therapy, and cancer vaccines, among various therapeutic approaches [20, 21, 22, 23, 24, 25, 26, 27]. Compared to traditional cancer treatments, immunotherapy has significantly mitigated the limitations of nonspecific cytotoxicity associated with chemotherapy and radiotherapy, while also overcoming resistance due to target mutations in targeted therapies. Thus, immunotherapy exhibits advantages of enhanced specificity, expanded therapeutic scope, and sustained clinical benefits [28, 29, 30, 31]. Numerous clinical studies have demonstrated that combining traditional treatment modalities with immunotherapy can significantly enhance treatment response rates and improve patient survival outcomes [8, 32, 33]. However, certain clinical trials have revealed inconsistent therapeutic effects [34], indicating that the synergistic mechanisms and optimal administration regimens of these combination therapies necessitate further investigation and elucidation.

The microbiome inhabiting the human body represents a complex ecosystem, consisting of various microbial communities including bacteria, fungi, viruses, and archaea, which are predominantly distributed throughout the oral cavity, respiratory system, gastrointestinal tract, reproductive system, and skin, exerting crucial impacts on human health [35]. Among these, the gastrointestinal microbiome represents the largest microbial ecosystem in the human body, harboring trillions of microbial cells [36]. The gut microbiome plays a vital role in maintaining the metabolic health of the human host; however, dysbiosis of the gut microbiome may promote the initiation and progression of various common metabolic disorders [35, 37, 38]. Beyond the gastrointestinal tract, the microbiome in other anatomical niches of the human body also plays key roles in maintaining local microenvironmental homeostasis and participating in pathological processes, including cancer [39, 40, 41, 42, 43, 44, 45, 46]. Additionally, research has identified a distinct class of microorganisms existing directly within tumor cells, defined as the intratumoral microbiome. The presence of microorganisms has been detected in tumor tissues across diverse malignancies, including oral cancer, hepatocellular carcinoma (HCC), colorectal cancer (CRC), pancreatic cancer, breast cancer, and melanoma [47]. These intratumoral microorganisms can either promote or inhibit tumor progression through multiple molecular mechanisms, thereby profoundly impacting human health [48, 49, 50]. In recent years, the intratumoral microbiome, as a crucial component of the tumor microenvironment (TME), has increasingly attracted widespread attention from the academic community, with research expanding from the exploration of fundamental biological mechanisms toward clinical diagnostic and therapeutic applications, thereby facilitating a deeper understanding among researchers of the complex interactions between the microbiome and human health.

The microbiome's influence on the host immune system constitutes one of the key mechanisms closely related to human health. Numerous studies have confirmed that the human microbiome not only contributes to immune microenvironment homeostasis but can also induce inflammatory responses and regulate antitumor immune responses. For instance, the gut microbiome has been demonstrated to participate in the maturation of the immune system and in the pathogenesis of immune‐related diseases [37, 51]. The microbiome plays a pivotal role in tumor initiation and progression, with immune system regulation serving as one of the key mediating mechanisms. Specifically, gut microbiome dysbiosis and abnormal bacterial composition within tumors are significantly associated with carcinogenesis and clinical outcomes [52, 53, 54]; viruses promote tumorigenesis by inducing immune evasion mechanisms [55, 56, 57]; whereas specific fungi primarily exert carcinogenic effects by activating inflammation‐related signaling pathways [58]. Based on the multidimensional regulatory effects of the microbiome on the immune system, its correlation with the efficacy of cancer immunotherapy rests upon a solid theoretical foundation, a hypothesis that has been substantiated by numerous experimental and clinical studies. Research indicates that the microbiome modulates the clinical efficacy of ICI and chimeric antigen receptor T‐cell (CAR‐T) cell therapies through diverse mechanisms, including regulating ICI target expression, influencing T cell activation, and altering cytokine secretion [59, 60, 61]. Additionally, specific microbial lineages or metabolites have been identified as potential biomarkers for predicting responses to cancer immunotherapy [62]. In clinical practice, microbial intervention strategies, including fecal microbiome transplantation (FMT), antibiotic (ATB) administration, and engineered bacteria derived from synthetic biology, have been demonstrated to significantly influence patient responses to immunotherapy, thereby providing robust support for bench‐to‐bedside translation and offering novel combination therapeutic approaches for cancer immunotherapy [63, 64, 65].

This review comprehensively examines the complex interaction mechanisms between cancer immunotherapy and the microbiome and their potential clinical applications, aiming to systematically integrate research progress from fundamental mechanisms to clinical translation while providing theoretical foundations and practical directions for the development of future microbiome‐targeted therapeutic strategies. This article thoroughly analyzes how the gut microbiome, intratumoral microbiome, and microbial communities from different ecological niches, including the oral cavity, skin, respiratory tract, and urogenital tract, regulate the tumor immune microenvironment, thereby influencing the clinical efficacy of ICIs and CAR‐T cell therapy. Existing research indicates that specific microbiome composition and diversity can not only predict patient responsiveness to immunotherapy but also directly enhance antitumor immune effects through various molecular mechanisms, including the regulation of T cell activity, the promotion of antigen presentation, and the remodeling of the TME. At the clinical application level, the combined use of microbiome regulation strategies—such as FMT, probiotic/prebiotic interventions, and synthetic biology‐modified engineered bacterial therapies—with immunotherapy has demonstrated significant synergistic therapeutic effects in various tumor types, thereby providing new solutions for overcoming primary and acquired resistance to immunotherapy. Despite current challenges, including the standardization of technical methodologies, the heterogeneity of clinical samples, and insufficient mechanistic insights, ongoing advancements in microbiome sequencing, single‐cell multiomics analysis technologies, and improved big data integration capabilities are expected to enable microbiome research to drive cancer immunotherapy toward more precise and personalized directions. This progress will provide solid scientific evidence for developing microbiome signature‐based precision treatment strategies, optimizing immunotherapy regimens, and improving long‐term patient outcomes, ultimately facilitating the realization of the clinical vision of integrating microbiome regulation strategies into modern comprehensive cancer immunotherapy systems.

## 2 Gut Microbiome and Cancer Immunotherapy

The gut microbiome, as the largest and most functionally complex microbial ecosystem in the human body, exerts profound influence on the development, homeostatic maintenance, and antitumor immune responses of the host immune system through its compositional and functional diversity. The compositional characteristics of the gut microbiome are intimately associated with the immune system, thereby establishing bidirectional interaction mechanisms that play pivotal roles in tumor immunotherapy responses. The associations between the gut microbiome and tumor immunotherapy are complex and multifaceted, and these fundamental insights provide the foundation for clinical translation of the gut microbiome in immunotherapeutic applications.

### 2.1 Composition and Functions of Gut Microbiome

Since their discovery in the 18th century, gut microorganisms have gradually had their biological complexities elucidated through continuous exploration by researchers. Gut microbiome is established at birth and develops during infancy, comprising bacteria, fungi, viruses, archaea, and protozoa [66, 67], and is influenced by various factors including host genetics, diet, ATBs, and psychological factors [68, 69]. As many as 100 trillion microbes are known to reside in the adult gut, and among these microorganisms, bacteria constitute the most abundant population [70]. *Bacteroidetes* and *Firmicutes* represent the primary gut microbiome phyla in humans, followed by *Actinobacteria*, *Fusobacteria*, *Proteobacteria*, and *Verrucomicrobia phyla* [67, 71]. Currently known gut fungi primarily encompass species from the *Candida*, *Saccharomyces*, and *Malassezia* [72, 73]. The human gut also harbors a large number of viruses, predominantly bacteriophages [74]. Archaea and protozoa presence in the gut microbiome has also been explored [75]. Gut microorganisms include probiotics and commensal fungi; the former encompass *Bifidobacterium*, *Lactobacillus*, and other beneficial bacteria, while the latter include *Candida albicans*, among others [72]. These microbial communities contribute to shaping the immune system, promoting nutritional and lipid metabolism, maintaining intestinal barrier homeostasis, and inhibiting colonization by pathogenic bacteria in the gut [70, 76]. Conversely, pathogenic bacteria, pathogenic fungi, gut viruses, and some commensal fungi can disrupt gut microbiome homeostasis, alter intestinal permeability, impair food and drug absorption, induce inflammatory diseases, and potentially lead to tumor development [70, 72, 77, 78, 79]. However, the microbiome cannot be simply classified into these two categories; when the host immune system is dysregulated, some commensal bacteria can convert into pathogenic forms, thereby compromising the host's health. Additionally, gut microorganisms produce diverse metabolites, which further influence intestinal health and microbial homeostasis.

### 2.2 Interaction Between Gut Microbiome and the Immune System

#### 2.2.1 Gut Microbiome Regulate Immune Cell Development and Distribution

**FIGURE 1:**
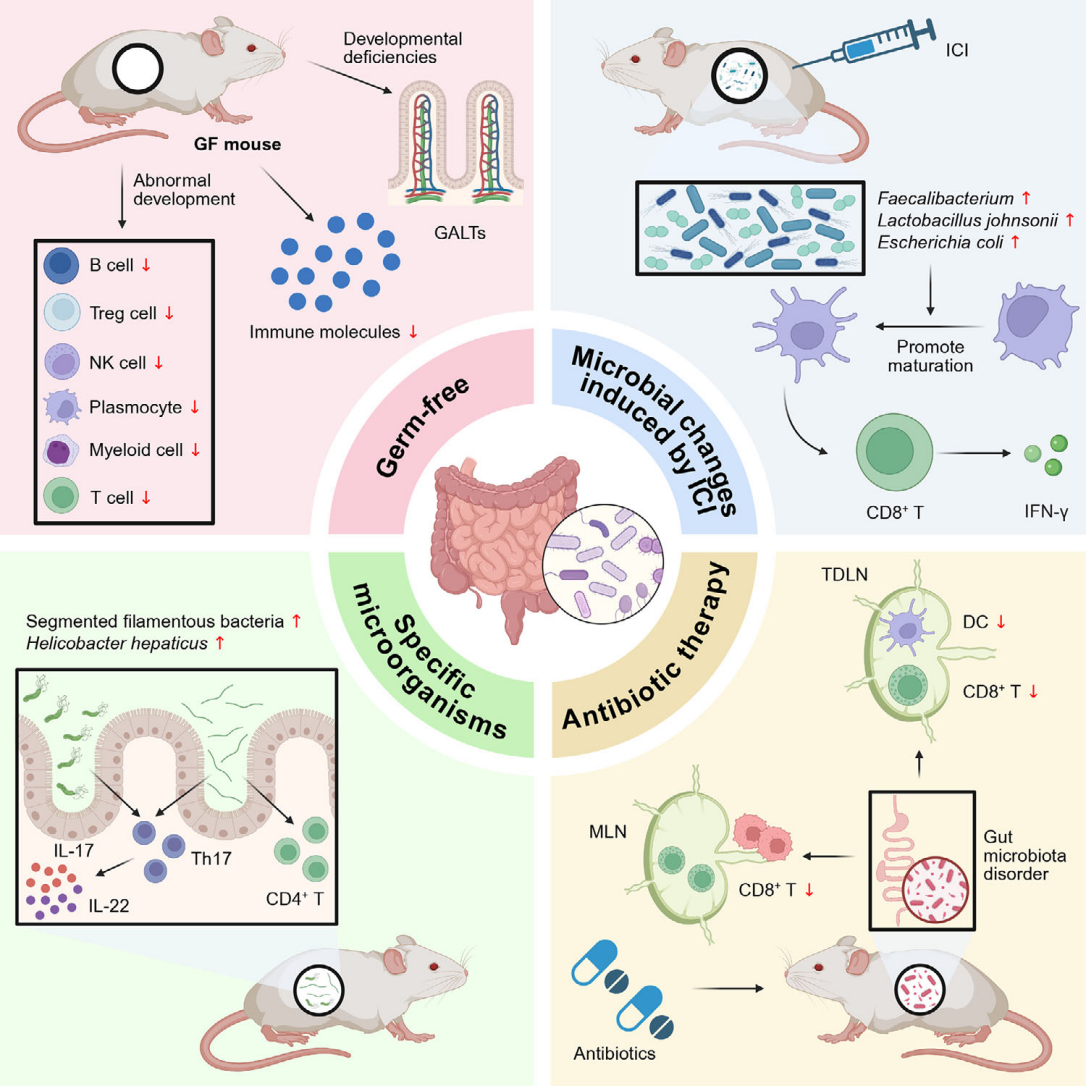
Critical role of gut microbiome in regulating immune cell development and distribution. The figure illustrates the abnormal development of the immune system in germ‐free mice, alongside microbial alterations following ICI therapy, microbial changes after ATB treatment, and the impact of specific bacterial communities on immune cell development and distribution in mice. This figure was created using the tools provided by Biorender.com (accessed on 08/04/2025). DC, dendritic cell; GALTs, gut‐associated lymphoid tissues; GF, germ‐free; ICI, immune checkpoint inhibitor; IFN‐γ, interferon‐γ; IL, interleukin; MLN, mesenteric lymph node; NK, natural killer; TDLN, tumor‐draining lymph node; Th17, T helper 17 cells.

The gut microbiome plays a crucial role in regulating immune cell development and distribution (Figure 1). Numerous animal experiments have confirmed that germ‐free mice exhibit significant immune system developmental deficiencies, primarily characterized by impaired development of gut‐associated lymphoid tissues (GALTs), which leads to a marked deficiency in secretory IgA [80, 81]. Furthermore, significant T cell developmental abnormalities have been observed in germ‐free mouse models [82]. Cross‐tissue multiomics analysis has revealed that microbiome‐deficient mouse models exhibit abnormal development of various immune cells, including B cells, myeloid cells, T cells, and natural killer (NK) cells. Compared to specific pathogen‐free (SPF) mice, these microbiome‐deficient mice demonstrate significantly reduced plasma cell aggregation areas, severely imbalanced adaptive immune cell proportions, and downregulation of multiple key immune molecules [83]. Further research has demonstrated that the gut microbiome significantly influences the developmental processes of both regulatory T cells (Tregs) and nonregulatory CD4^+^ T cells [84]. Specific commensal microorganisms (including segmented filamentous bacteria and *Helicobacter hepaticus*) specifically induce T cell subpopulations that recognize commensal bacteria by regulating T cell differentiation processes [85]. Notably, specific microorganisms such as segmented filamentous bacteria promote the development and maturation of specific T cell subpopulations in the thymus [86]. Research has demonstrated that the microbiome alterations induced by immune checkpoint blockade (ICB) treatment (primarily manifested as marked increases in bacterial groups such as *Enterococcus faecalis*, *Lactobacillus johnsonii*, and *Escherichia coli*) could, in turn, induce dendritic cell (DC) maturation, subsequently promoting the activation and proliferation of interferon‐γ (IFN‐γ)‐secreting CD8^+^ T cells [87]. Gut microbial dysbiosis caused by ATB therapy significantly reduces the number of activated DCs in tumor‐draining lymph nodes (TDLNs), decreases the density of effector CD8^+^ T cells in mesenteric lymph nodes (MLNs) and TDLNs, and ultimately weakens the host's antitumor immune response [87]. Gut microbiome‐derived hyocholic acid promotes the development of γ–δ T cells and type 3 innate lymphoid cells (ILC3), which play a crucial role in the development of type 3 immune responses in the neonatal intestine [88].

#### 2.2.2 Gut Microbiome Influence Immune Cell Function and Regulate Cytokine Expression

Gut microbiome also influences immune responses within the TME through various regulatory mechanisms, including activation of pattern recognition receptors, molecular mimicry, and microbiome‐derived metabolites [89]. Research has established that various bacterial groups, including *Escherichia coli Nissle 1917 strain*, *ECOR12 strain*, *Bifidobacterium*, and *Lactobacillus paracasei*, significantly regulate the functions of multiple immune cells in the intestine, such as DCs, CD4^+^ T lymphocytes, T helper 1 cell (Th1) effector cells, and Treg cells, and modulate the expression of key cytokines, including IFN‐γ, interleukin (IL)‐12, IL‐10, and transforming growth factor‐β (TGF‐β), thereby comprehensively affecting the balance of the intestinal immune system [90, 91, 92]. Gut microbiome metabolites, such as short‐chain fatty acids (SCFAs), tryptophan metabolites, and polyamines, regulate immune cell functions [93]. For example, gut microbiome dysbiosis exacerbates the regulatory effects of tryptophan by modulating specific macrophage functions [94]. Additionally, microbial‐derived B vitamins also regulate local and systemic immune responses [95]. Research has demonstrated that polysaccharide A, a metabolite of gut *Bacteroides fragilis*, increases Treg abundance, thereby inducing a tolerogenic environment [96]. Notably, SCFAs regulate the functions of various immunosuppressive cells, including Tregs, Bregs, DCs, and IL‐10‐producing macrophages, which play critical roles in suppressing inflammatory responses [97]. Gut microbial metabolites with immunosuppressive functions have been confirmed to be closely related to the inhibition of ferroptosis processes, while gut microbial metabolites with proinflammatory characteristics accelerate ferroptosis. Numerous studies have confirmed the close association between ferroptosis processes and tumor development; therefore, by selectively regulating the composition of gut microbiome and the distribution and concentration of their metabolites in the intestinal microenvironment, precise regulation of immune cell ferroptosis processes can be achieved, thereby inducing a microenvironment favorable for antitumor immune responses [98]. Furthermore, bacterial extracellular vesicles (BEVs) regulate the function and activity of immune cells in the intestine by precisely controlling the balance between T helper 17 cells (Th17) and Tregs and influencing cytokine secretion.

#### 2.2.3 Immune System Regulates and Maintains Intestinal Microbial Homeostasis

One of the key functions of the intestinal immune system is to distinguish between commensal and pathogenic bacteria, primarily mediated by Toll‐like receptors (TLRs), to maintain intestinal barrier stability [99]. The immune system simultaneously influences the composition and abundance of the gut microbiome. For instance, intestinal T cells sense microbial signals and regulate immunoglobulin A (IgA) secretion through the myeloid differentiation factor 88 (MyD88) signaling pathway, while high‐affinity IgA targeting commensal bacteria further maintains gut microbiome homeostasis. Deficiency in this signaling pathway leads to dysregulated control of the microbial community composition, subsequently triggering intestinal diseases [100]. Activation of intestinal immune responses rapidly alters the metabolic characteristics of the gut microbiome. Specifically, the activated immune system rapidly modifies the transcriptome and metabolome of the gut microbiome, inhibits the production of SCFAs (especially acetate), and reduces SCFA concentrations in the intestinal microenvironment, thereby affecting host immune responses or inducing disease [101]. Research demonstrates that during immunotherapy of oral squamous cell carcinoma (MOC2) mice, those with favorable treatment responses exhibit higher levels of *Romboutsia*, *Turicibacter*, and *Peptococcus*, while mice with poor therapeutic efficacy show the gut microbiome mainly enriched with *Enterorhabdus*, *Desulfovibrio*, *Streptococcus*, and *Staphylococcus* [102]. Intestinal DCs and macrophages recognize microbiome‐derived antigens that traverse the intestinal epithelial cell (IEC) barrier and present them to B and T cells, thereby regulating adaptive immune responses [103]. Furthermore, GALT relies on the gut microbiome to promote the production of IgA‐secreting plasma cell precursors, thereby preserving the homeostasis of the intestinal microenvironment [104].

#### 2.2.4 Gut Microbiome–Immune System Interactions Promote Disease Development

The gut microbiome significantly influences the development and function of T cells, Tregs, and natural killer T cells (NKT), with these effects intricately associated with various immune‐related diseases, including rheumatoid arthritis, inflammatory bowel disease (IBD), allergic asthma, and multiple cancers [93, 105, 106]. Research reveals that intestinal IgA deficiency induces dysbiosis of gut microbiome, subsequently triggering systemic inflammatory responses in patients with primary antibody deficiency, with *Enterococcus* identified as a potential pathogenic factor in common variable immunodeficiency disease‐associated immune dysregulation complications (CVIDid) [107]. Gut microbiome exerts procarcinogenic effects through multiple mechanisms, including the induction of inflammatory responses, promotion of immunosuppression, and metabolic production of toxic substances, leading to genomic damage and immune evasion in tumor tissues. Conversely, other studies demonstrate that specific intestinal microbes reshape the TME, exhibiting inhibitory effects on tumor progression [108, 109]. Gut microbiome also promotes cytokine production through TLR signaling pathways, subsequently activating the nuclear factor κB (NF‐κB) signaling pathway and inducing inflammatory responses. Numerous studies confirm that activation of the NF‐κB signaling pathway induced by TLRs (especially TLR4) plays a crucial role in the development and progression of various malignancies, including CRC, pancreatic cancer, and HCC [110, 111]. Gut microbiome not only affects the digestive system but also exerts significant regulatory effects on other tissues and organs, particularly the nervous system. In recent years, the microbiome–gut–brain axis has emerged as a critical research focus, attracting widespread attention from the scientific community. Recent explorations of the gut microbiome–immune–brain axis reveal that interactions between gut microbiome and the immune system significantly influence the development and function of the nervous system, with these effects intimately associated with the pathogenesis of various neurological disorders, including major depressive disorder, schizophrenia, multiple sclerosis, Parkinson's disease, and Alzheimer's disease [112]. Simultaneously, the nervous and immune systems bidirectionally modulate the composition and homeostasis of the gut microbiome through neuroendocrine and immunomodulatory mechanisms, forming a complex regulatory network [112].

### 2.3 Mechanisms by Which Gut Microbiome Influence the Efficacy of Cancer Immunotherapy

The gut microbiome significantly influences cancer immunotherapy by regulating the immune system, particularly affecting the efficacy of ICIs and CAR‐T therapy. The gut microbiome is closely associated with ICI treatment responses, with specific microbial communities enhancing antitumor effects through immune activation, while dysbiosis may attenuate therapeutic responses. Additionally, the gut microbiome can regulate immune cell functions by producing metabolites, reshaping the TME, thereby influencing ICI efficacy. Characteristics of the gut microbiome significantly affect the prognosis of immunotherapy patients, with certain microbial communities potentially exacerbating treatment‐related adverse events, suggesting that modulating the gut microbiome may represent a novel strategy for optimizing ICI efficacy and improving prognosis. Furthermore, the association between gut microbiome and CAR‐T therapy has been extensively explored and elucidated.

#### 2.3.1 Gut Microbiome Associated With ICI Treatment Response

The gut microbiome has been confirmed to be closely associated with ICI treatment responses in various cancers (Table 1), including melanoma, non–small‐cell lung cancer (NSCLC), renal cell carcinoma (RCC), HCC, and gastrointestinal cancers [113]. A study focusing on melanoma patients revealed significant differences in intestinal microbial diversity and composition between responders and nonresponders to anti‐PD‐1 immunotherapy. Specifically, responders exhibited significantly higher α diversity and notable enrichment of *Clostridiales*, *Ruminococcaceae*, and *Faecalibacterium* genus, which may be associated with increased expression of effector CD4^+^ and CD8^+^ T cells, as well as enhanced cytokine responses crucial for maintaining anti‐PD‐1 therapeutic effects [114]. In contrast, nonresponders showed significantly increased abundance of *Bacteroides thetaiotaomicron*, *Escherichia coli*, and *Anaerotruncus colihominis*, which may be closely associated with upregulation of Tregs and myeloid‐derived suppressor cells (MDSCs), as well as downregulation of cytokine responses [114]. Another study further confirmed that responders exhibited significantly higher abundance of *Bifidobacterium longum*, *Collinsella aerofaciens*, and *Enterococcus faecium* compared to nonresponders, with these microbial differences resulting in decreased exogenous Tregs, increased Batf3‐lineage DCs, and enhanced Th1 responses [115]. This research found that transplanting fecal microbiome from responders into germ‐free mice significantly improved the efficacy of antiprogrammed cell death‐ligand 1 (PD‐L1) therapy [115]. Similar research findings were observed in lung cancer (LC) and renal cancer patients, where responders to ICI therapy exhibited significantly higher levels of *Akkermansia muciniphila* (*AKK*) compared to nonresponders, suggesting that anti‐PD‐1 therapy may induce Th1 immune responses, thereby enhancing cancer immunosurveillance [116]. Additionally, studies on NSCLC patients receiving anti‐PD‐1 therapy found that responders exhibited significantly higher bacterial diversity and notably increased abundance of *Alistipes putredinis*, *Bifidobacterium longum*, and *Prevotella copri*, while nonresponders showed significant enrichment of *Ruminococcus_unclassified* [117]. Results from multiomics analyses revealed that 11 bacterial genera were significantly positively correlated with anti‐PD‐1/PD‐L1 treatment responses, including *Butyricicoccus*, *Adlercreutzia*, and *Allisonella*, while seven genera showed significant negative correlations, including *Klebsiella* and *Erysipelatoclostridium*. This study further identified five intestinal microbial community types (enterotypes) associated with treatment responses, each with specific microbial compositions and metabolite characteristics. Notably, the study found that the metabolite phenylacetylglutamine (PAGln) was significantly associated with poor anti‐PD‐1 efficacy [118]. Another study focusing on elderly patients revealed that the E/AE enterotype was significantly positively correlated with high responsiveness to ICB therapy, with this association likely mediated by differences in the gut microbiome composition [119]. Specifically, *AKK*, *Parabacteroides merdae*, and *Alistipes putredinis* in the enterotype/aging‐enriched (E/AE) enterotype were significantly associated with enhanced antitumor efficacy, while *B. thetaiotaomicron* and *B. fragilis* were significantly associated with inhibitory effects on CTLA‐4 blockade resistance [119]. Furthermore, in mouse models receiving FMT from the E/AE enterotype, researchers identified five significantly enriched bacteria, including *Bilophila wadsworthia*, *Bacteroides ovatus*, and *B. uniformis*, which are considered potential beneficial microbes for promoting ICB treatment responses [119]. In conclusion, the gut microbiome can significantly regulate immunotherapy efficacy, with numerous recent studies dedicated to exploring the key molecular mechanisms involved.

**TABLE 1:** Association between gut microbiome composition and response to ICI therapy across diverse cancer types.

Cancer type	Immunotherapy	Clinical significance	Microbial changes	Mechanism	References
Melanoma	Anti‐PD‐1 immunotherapy	Response	*Clostridiales* ↑ *Ruminococcaceae* ↑ *Faecalibacterium* ↑	Increased expression of effector CD4^+^ and CD8^+^ T cells; Enhanced cytokine responses sustain anti‐PD‐1 therapy.	[114]
	Anti‐PD‐1 immunotherapy	Nonresponse	*Bacteroides thetaiotaomicron* ↑ *Escherichia coli* ↑ *Anaerotruncus colihominis* ↑	The number of Tregs was upregulated; Upregulation of MDSC numbers; Downregulation of cytokine responses.	
Metastatic melanoma	Anti‐PD‐1 immunotherapy	Response	*Bifidobacterium longum* ↑ *Collinsella aerofaciens* ↑ *Enterococcus faecium* ↑	Exogenous Tregs were reduced; Increased numbers of Batf3‐lineage DCs; Enhanced Th1 responses.	[115]
Lung cancer Renal cell carcinoma	Anti‐PD‐1 immunotherapy	Response	*Akkermansia muciniphila* ↑	Recruitment of CCR9^+^/CXCR3^+^/CD4^+^ T lymphocytes.	[116]
NSCLC	Anti‐PD‐1 immunotherapy	Response	*Alistipes putredinis* ↑ *Bifidobacterium longum* ↑ *Prevotella copri* ↑	The frequency of memory CD8^+^ T cells was increased; A higher frequency of NK cell subsets.	[117]
	Anti‐PD‐1 immunotherapy	Nonresponse	*Ruminococcus_unclassified* ↑		
Colorectal cancer GC RCC NSCLC	Anti‐PD‐1 immunotherapy	Response	*Butyricicoccus* ↑ *Adlercreutzia* ↑ *Allisonella* ↑ *Candidatus* ↑ *Clostridiales* ↑ *Klebsiella* ↓ *Erysipelatoclostridium* ↓		[118]
	Anti‐PD‐1 immunotherapy	Response	Microbial metabolite PAGln ↑	Reduction of cytotoxic CD8^+^ T cells.	
Pan‐cancer	ICB	Increased efficacy	*Bacteroides thetaiotaomicron* ↑ *Bacteroides fragili*s ↑ *Akkermansia muciniphila* ↑ *Parabacteroides merdae* ↑ *Alistipes putredinis* ↑		[119]

Abbreviations: ↑, increased; ↓, decreased; DCs, dendritic cells; GC, gastric cancer; ICB, immune checkpoint blockade; ICI, immune checkpoint inhibitor; MDSC, myeloid‐derived suppressor cell; NK, natural killer; NSCLC, non–small‐cell lung cancer; PAGln, phenylacetylglutamine; RCC, renal cell carcinoma; Th1, helper T‐cell 1; Tregs, regulatory T cells.

#### 2.3.2 Mechanisms by Which Gut Microbiome Influence the Efficacy of ICI Therapy

**FIGURE 2:**
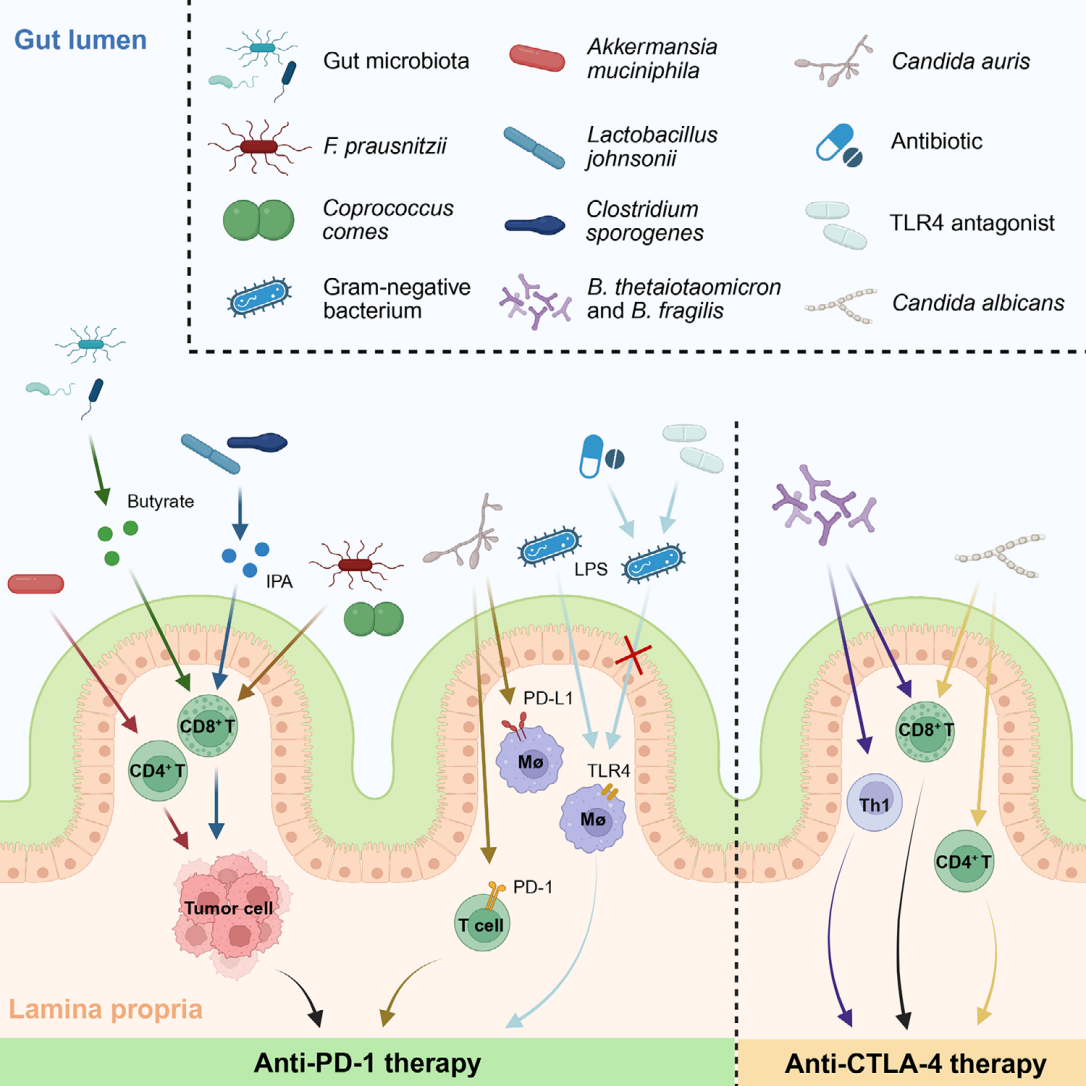
Regulatory effects of gut microbiome on ICI therapy. This figure depicts various bacteria and fungi that influence the efficacy of anti‐PD‐1 and anti‐CTLA‐4 therapies by regulating diverse immune processes, including CD4^+^ T, CD8^+^ T, macrophage, and Th1 cell activation and infiltration. This figure was created using the tools provided by Biorender.com (accessed on 08/04/2025). CTLA‐4, cytotoxic T‐lymphocyte‐associated antigen 4; ICI, immune checkpoint inhibitor; IPA, indole‐3‐propionic acid; LPS, lipopolysaccharide; PD‐1, programmed cell death protein 1; PD‐L1, programmed cell death‐ligand 1; Mø, macrophages; Th1, T helper 1 cell; TLR4, Toll‐like receptor 4.

The gut microbiome profoundly influences the therapeutic efficacy of ICIs via the aforementioned fundamental immune regulatory mechanisms. This regulatory function involves complex interactions among the microbial community itself, its metabolites, and the host immune system. The regulatory role of the intestinal microbiome in ICI therapy is manifested in multiple aspects, involving complex interactions between various bacteria and the host immune system [38, 120] (Figure 2). Research indicates that the microbiome primarily regulates host immune responses through three fundamental mechanisms: chemokine release, molecular mimicry, and immunometabolic regulation [59]. Numerous studies have confirmed that the gut microbiome can promote CD8^+^ T cell‐mediated immune responses by activating and stimulating DCs, which serve as critical antigen‐presenting cells [121]. Specifically, *B. thetaiotaomicron* or *B. fragilis* can significantly affect the therapeutic effect of CTLA‐4 blockers by promoting the Th1 response and activating CD8^+^ T cells [122]. Characteristic microbes significantly enriched in various cancer patients (including *Faecalibacterium prausnitzii*, *Coprococcus comes*, etc.) can influence ICI treatment efficacy by modulating CD8^+^ T cell activity, a phenomenon that has been confirmed in both in vitro experiments and mouse models [123]. Furthermore, *AKK* can promote the recruitment and infiltration of CD4^+^ T cells into the TME, enhance immune surveillance functions, and inhibit tumor cell immune escape mechanisms, ultimately improving the clinical efficacy of PD‐1 blockade immunotherapy [116].

The intestinal microbiome can not only enhance ICI efficacy by regulating host immune responses but also further improve immunotherapeutic outcomes by producing various bioactive metabolites (including SCFAs, tryptophan metabolites, and inosine) [89]. Research has found that *Lactobacillus johnsonii* and *Clostridium sporogenes* in the intestinal commensal microbiome work synergistically to produce the tryptophan metabolite indole‐3‐propionic acid (IPA), which can significantly promote CD8^+^ T cell infiltration into tumor tissues, thereby enhancing the efficacy of anti‐PD‐1 immunotherapy [124]. Experimental studies in animal models have shown that the efficacy of anti‐PD‐1 therapy in tumor‐bearing mice largely depends on microbiome‐derived hexa‐acylated lipopolysaccharide (LPS) [125]. Orally administered hexa‐acylated LPS can significantly enhance anti‐PD‐1 efficacy, while the combined use of ATBs or small‐molecule TLR4 antagonists can significantly inhibit the antitumor effect of PD‐1 blockade, further confirming the critical nature of this mechanism [125]. Additionally, research has confirmed that various gut microbiome metabolites can significantly improve ICI efficacy, primarily attributable to their diffusion capacity in vivo and regulatory effects on local and systemic antitumor immune responses [126, 127]. For example, butyrate, a metabolite produced by the gut microbiome, can significantly promote CD8^+^ T cell‐mediated immune responses, thereby enhancing the efficacy of antitumor immunotherapy [128]. Meanwhile, microbial‐produced metabolite butyrate inhibits the expression levels of PD‐L1 and IL‐10. These effects on the immune system not only inhibit tumor development but also promote the reversal of immunosuppression.

Beyond bacteria, the intestinal mycobiome also demonstrates important biological associations with ICI efficacy, representing another dimension of microbiome influence. Research indicates that *Candida albicans* can promote the activation and recruitment of CD4^+^ T cells and CD8^+^ T cells to tumor sites by modulating the CTLA‐4 signaling pathway [129]. *Candida auris* infection can induce upregulation of PD‐1/PD‐L1 expression, a mechanism that potentially enhances the clinical efficacy of anti‐PD‐1/PD‐L1 therapy [130].

#### 2.3.3 Gut Microbiome Influences Prognosis and Treatment‐Related Adverse Events in Patients Receiving ICI Therapy

The gut microbiome is not only closely associated with the efficacy of ICIs, but also substantially influences long‐term patient prognosis and plays a crucial role in the development of immune‐related adverse events (irAEs) [131]. Studies have demonstrated that during ICB therapy, patients with progression‐free survival (PFS) less than 12 months exhibit a significant increase in proinflammatory bacterial communities, primarily including species such as *Bacteroides clarus*, *Streptococcus intestinalis*, and *Enterococcus tayi*. However, it is noteworthy that due to the heterogeneity of clinical research settings, significant enrichment of certain proinflammatory bacterial communities (such as *Parabacteroides merdae*, *Desulfovibrio piger*, and *Streptococcus oralis*) has also been observed in long‐term benefit patients with PFS ≥ 12 months [132]. Furthermore, genomic structural variations of gut microorganisms such as *AKK*, *Dorea formicigenerans*, and *Bacteroides* have been found to exhibit significant correlations with clinical efficacy and long‐term prognosis of ICI therapy [133].

ICI therapy frequently induces irAEs, and accumulating evidence indicates that gut microbial composition is closely associated with the underlying mechanisms and severity of these adverse reactions. Research has observed that approximately 10 days prior to the onset of immune therapy‐related colitis (irC), patients’ gut microbial communities exhibit significant changes, primarily characterized by markedly reduced microbial diversity, increased abundance of *Proteobacteria* and *Veillonella*, and decreased *Faecalibacterium*; interestingly, these microbial composition features tend to normalize before the resolution of irC symptoms [134]. Growing experimental and clinical evidence suggests a causal relationship between gut commensal microbiome and the initiation, progression, and severity of irAEs [135]. Studies have confirmed that patients with baseline gut microbiome enriched in *Faecalibacterium* and other members of *Firmicutes* not only demonstrate more favorable clinical responses to ipilimumab treatment, but also exhibit significantly higher rates of ipilimumab‐induced colitis [136]. Additionally, clinical research data indicate that prolonged use of broad‐spectrum ATBs not only significantly increases the risk of ICI‐induced colitis but also substantially exacerbates the severity of its clinical manifestations [137].

#### 2.3.4 Gut Microbiome Influences the Efficacy and Prognosis of CAR‐T Therapy

Furthermore, studies have revealed that specific gut microbial compositions significantly influence the clinical efficacy of CAR‐T immunotherapy [60]. A recent study confirmed that specific fecal bacterial communities, including *Ruminococcus*, *Faecalibacterium*, and *Bacteroides*, are associated with CD19 CAR‐T cell therapy response, with *Ruminococcus* and *Faecalibacterium* genera positively correlating with complete response at Day 100 [138], while the order *Veillonellales* and family *Veillonellaceae* are significantly associated with reduced CR at Day 100 [138]. Microbial communities, notably the *Bifidobacterium genus*, can directly regulate CAR‐T cell activity and may also modulate the severity of CAR‐T therapy‐related cytokine release syndrome by regulating the activity of macrophages and monocytes in recipients, thereby leading to alterations in IL‐1 and IL‐6 cytokine concentrations [139, 140]. Animal experiments have demonstrated that microbial metabolites, specifically SCFAs, can regulate CD8^+^ T cell responses and enhance the metabolic adaptability of CAR‐T cells, thereby improving their cytotoxic effects against tumor cells [141]. Administration of ATBs targeting anaerobic commensals (meropenem, cefepime, ceftazidime, and piperacillin‐tazobactam) to lymphoma patients prior to CAR‐T cell therapy has been shown to lead to gut microbiome dysbiosis and metabolite secretion imbalance, potentially resulting in reduced efficacy of anti‐CD19 CAR‐T therapy, as evidenced by lower PFS and overall survival (OS) [142, 143]. OVs represent an effective antitumor immunotherapeutic strategy; however, recent research has discovered that the OV VSVIFNβ can cause depletion of CAR‐T cells and conventional T cells through IFN expression, suppressing antitumor activity and hindering the formation of combined treatment strategies [144]. Although the association between the microbiome and CAR‐T therapy has been widely reported, the underlying mechanisms remain insufficiently explained and require further investigation to explore potential mechanisms.

### 2.4 Gut Microbiome as Potential Targets for Cancer Immunotherapy

Therapeutic strategies targeting gut bacteria can modulate the TME and stimulate the immune system while specifically targeting tumor cells, and are therefore considered cancer immunotherapies with enormous potential. Specifically, attenuated bacteria induce and activate antitumor responses by retaining their immunostimulatory properties, wherein attenuated *Salmonella*, *Listeria monocytogenes*, and *Mycobacterium bovis* Bacillus Calmette–Guerin (BCG) have all demonstrated significant anticancer activity [63]. Genetically engineered bacteria (such as engineered *Listeria monocytogenes* and engineered *Escherichia coli*), OVs, and modified bacteriophages can also stimulate the immune system, thereby promoting antitumor responses [63]. Engineered *Salmonella enterica* strain can inhibit the proliferation and metastasis of various tumors by inducing tumor‐associated macrophages to secrete IL‐10 and stimulating CD8^+^ T cells, while evading phagocytosis by tumor‐associated neutrophils. Tumor‐targeting bacteria can be engineered to continuously, specifically, and exclusively release IL‐10 within tumor cells, thereby becoming potential immunotherapeutic targets [145]. Studies indicate that various gut microorganisms (such as *Bifidobacterium*) play crucial roles in the efficacy of anti‐CTLA‐4 and anti‐PD‐L1 treatments, while ATB‐treated mice exhibit significantly reduced responses to ICI therapy [60]. These studies provide scientific evidence for employing gut microbiome modulation as a potential target for cancer immunotherapy, suggesting that targeting the gut microbiome may become an important strategy for cancer immunotherapy in the future.

CRC‐related research demonstrates that oral reovirus enhances antitumor immunity by reshaping the gut microbiome; although it cannot directly infect tumors outside the gastrointestinal tract, it can promote T cell activation, antigen presentation, and induction of type I/II interferon secretion in distant tumors [146]. This mechanism depends on the synergistic action of the gut microbiome, Batf3 DCs, and CD8^+^ T cells, and when combined with αPD‐1/αCTLA‐4, it can achieve complete regression of colon tumors [146]. The regulatory effects of specific bacterial communities have also been confirmed. *Faecalibacterium*, *Ruminococcaceae*, and *Bifidobacteriaceae* communities positively correlate with ICI efficacy, while ATB pretreatment disrupts the recovery of these key bacterial communities [147]. At the mechanistic level, tumor microbiome can train the immune system through bacterial antigen presentation, enabling T cells to recognize tumor cells as being in an “infected” state, while bacterial peptides may serve as neoantigens to enhance immune responses [131]. Preclinical trials have further verified that a mix of four *Clostridiales* strains can induce CD8^+^ T cell infiltration into melanoma and CRC tissues [148], while the *Ruminococcaceae* preparation SER401 combined with anti‐PD‐1 therapy has been demonstrated to be safe in clinical applications [147]. These findings collectively reveal that enhancing antitumor immune responses through targeted regulation of the gut microbiome has become an important strategic direction for cancer immunotherapy.

## 3 Intratumoral Microbiome and Cancer Immunotherapy

Beyond the gut microbiome, microorganisms colonizing tumor tissues (including bacteria, fungi, and viruses) have recently been discovered to play critical roles in tumorigenesis, progression, and immunotherapy responses. Intratumoral microbiome can participate in tumor immune regulation through mechanisms including local modulation of the immune microenvironment, modulation of antigen presentation, and modulation of inflammatory responses. The taxonomic characteristics of the intratumoral microbiome, their interactions with the immune microenvironment, and their potential therapeutic applications in immunotherapy are garnering increasing attention.

### 3.1 Types and Characteristics of Intratumoral Microbiome

#### 3.1.1 Intratumoral Bacteria

Intratumoral microbiome has been extensively studied, with current research having identified various microorganisms including bacteria, fungi, and viruses in multiple tumor tissues that exhibit significant differences in abundance and compositional diversity compared to normal tissues. Specifically, bacterial abundance in tumor tissues exhibits significant alterations. For instance, studies indicate that bacterial concentrations in breast tumors are 10‐fold higher compared to normal tissues, yet microbial diversity is decreased, with significant increases in *Methylobacterium radiotolerans* and notable reductions in the abundance of *Sphingomonas yanoikuyae*, *Anaerococcus*, and *Corynebacterium* [43, 149, 150]. Notably, functional in vitro studies have demonstrated that *Escherichia coli* and *Staphylococcus epidermidis* isolated from breast cancer patients can induce DNA double‐strand breaks in HeLa cells (human cervical cancer cells), thereby triggering chromosomal instability and promoting cancer progression [150]. Moreover, the intratumoral microbiome exhibits distinct cancer‐specific heterogeneity characteristics, with different tumor types harboring specific microbial compositional profiles. For example, the predominant bacteria in breast tumor tissues are *Proteobacteria*, *Firmicutes*, *Actinobacteria*, *Cyanobacteria*, and *Bacteroidetes*; while in CRC, *Firmicutes* and *Bacteroidetes* are most common; pancreatic cancer is characterized by a dominant bacterial community of *Proteobacteria*, a composition that closely resembles the normal duodenal microbiome, potentially reflecting the retrograde migration of duodenal bacteria into the pancreas through the pancreatic duct; and *Corynebacteriaceae* and *Micrococcaceae* family bacteria are primarily enriched in nongastrointestinal tumors [151, 152]. Notably, breast cancer tissues exhibit richer microbial diversity compared to other tumor types, and significant microbial profile differences exist between different molecular subtypes and pathological manifestations of breast tumors [152]. Specifically, studies have revealed that *Burkholderia* bacteria exhibit significant positive correlations with invasive triple‐negative breast cancer (TNBC) and basal‐like breast tumors. Conversely, genera such as *Alkanindiges*, *Anoxybacillus*, and *Leifsonia* demonstrate stronger associations with HER2‐enriched breast tumors. Notably, *Alkanindiges* shows a negative correlation with lymphovascular invasion, a finding that may be related to the aggressive nature and poor prognosis of HER2‐enriched breast cancer. Furthermore, *Citrobacter* demonstrates significant enrichment in advanced breast tumors [150]. In contrast, NSCLC microbiological characteristics are characterized by significant enrichment of *Romboutsia*, *Novosphingobium*, *Acinetobacter*, and *Prevotella* genera [153].

#### 3.1.2 Intratumoral Fungi

Research indicates that almost all tumor types (including breast cancer, colon cancer, pancreatic cancer, LC, and melanoma) exhibit increased fungal abundance, with common fungal groups including *Candida albicans*, *Malassezia*, and *Saccharomyces cerevisiae* [154]. In the context of pancreatic cancer, fungi contribute to tumor progression through complement cascade activation and IL‐33 secretion. Recent studies show that fungal communities in head and neck, colon, rectal, gastric, and esophageal tumors exhibit high similarity, while fungal compositions in nongastrointestinal tumors demonstrate significant differences, which may be related to tissue microenvironment pH, oxygen availability, and bacterial community composition [155]. Notably, *yeasts* (especially *Saccharomyces cerevisiae*) exhibit significant enrichment across various tumors, while highly enriched *Candida* in gastric adenocarcinoma has been confirmed to be closely associated with the development of early gastric adenocarcinoma [48, 155]. Furthermore, fungal concentrations in pancreatic tumor tissues are up to 3000‐fold higher than in normal pancreatic tissues, with significant enrichment of *Malassezia* observed in both human and mouse models [156]. Pathogenic fungi in pancreatic cancer may activate the complement cascade via mannose‐binding lectin (MBL)‐C3 pathway activation, thereby promoting pancreatic cancer development. Similarly, significant enrichment of *Malassezia* has also been detected in melanoma [48].

#### 3.1.3 Intratumoral Viruses

The composition of the intratumoral microbiome also includes a diverse array of viruses with well‐established carcinogenic effects. Well‐characterized oncogenic viruses include human papillomavirus (HPV) in cervical cancer, hepatitis B virus (HBV) and hepatitis C virus (HCV) in HCC, Epstein–Barr virus (EBV) in nasopharyngeal carcinoma (NPC) and lymphoma, human herpesvirus type 8 (HHV‐8) in Kaposi's sarcoma, human T‐cell leukemia virus type 1 (HTLV‐1) in adult T‐cell leukemia/lymphoma (ATLL), Merkel cell polyomavirus (MCPyV) in Merkel cell carcinoma, human polyomaviruses (including JCPyV and BKPyV) in Hodgkin lymphoma, and human immunodeficiency virus (HIV) [49]. These oncogenic viruses primarily induce tumorigenesis through several key mechanisms: integration of viral DNA into the host genome, interference with cell cycle regulation, promotion of immune‐inflammatory factor dysregulation, and upregulation of oncogenic signaling pathways. Specifically, high‐risk HPV types, particularly HPV16 and HPV18, are frequently detected in cervical cancer tissues; these viruses promote tumor development and progression by integrating their viral genome into the host cell genome [157]. Similarly, HBV, MCPyV, and human polyomaviruses exert their oncogenic effects by integrating viral DNA into the host cell genome, thereby generating tumor‐promoting mutant viral proteins and modulating tumor‐associated signaling pathways [49, 158, 159]. Research demonstrates that HCV can induce tumor‐promoting inflammatory responses, facilitate epigenetic modifications, and enhance oxidative stress processes, while HIV primarily exerts carcinogenic effects by suppressing host immune function and inducing genetic alterations, with both viruses interfering with the host immune system [160, 161]. Following EBV infection, the proliferation regulatory mechanisms of B lymphocytes become dysregulated, transforming these cells into lymphoblasts with unlimited proliferation potential, thereby significantly increasing the risk of carcinogenesis [162, 163]. Both HTLV‐1 and HHV‐8 can activate the NF‐κB signaling pathway, consequently promoting tumor development and progression. Notably, specific viruses are frequently associated with multiple tumor types: HPV is strongly related to oral cancer development in addition to cervical cancer; EBV is not only associated with NPC and lymphoma but also demonstrates a significant correlation with gastric cancer (GC) development; and HIV infection can promote the occurrence of various tumors including Kaposi's sarcoma, lymphoma, and non‐Hodgkin lymphoma [49, 164].

### 3.2 Impact of Intratumoral Microbiome on the Tumor Immune Microenvironment

**FIGURE 3:**
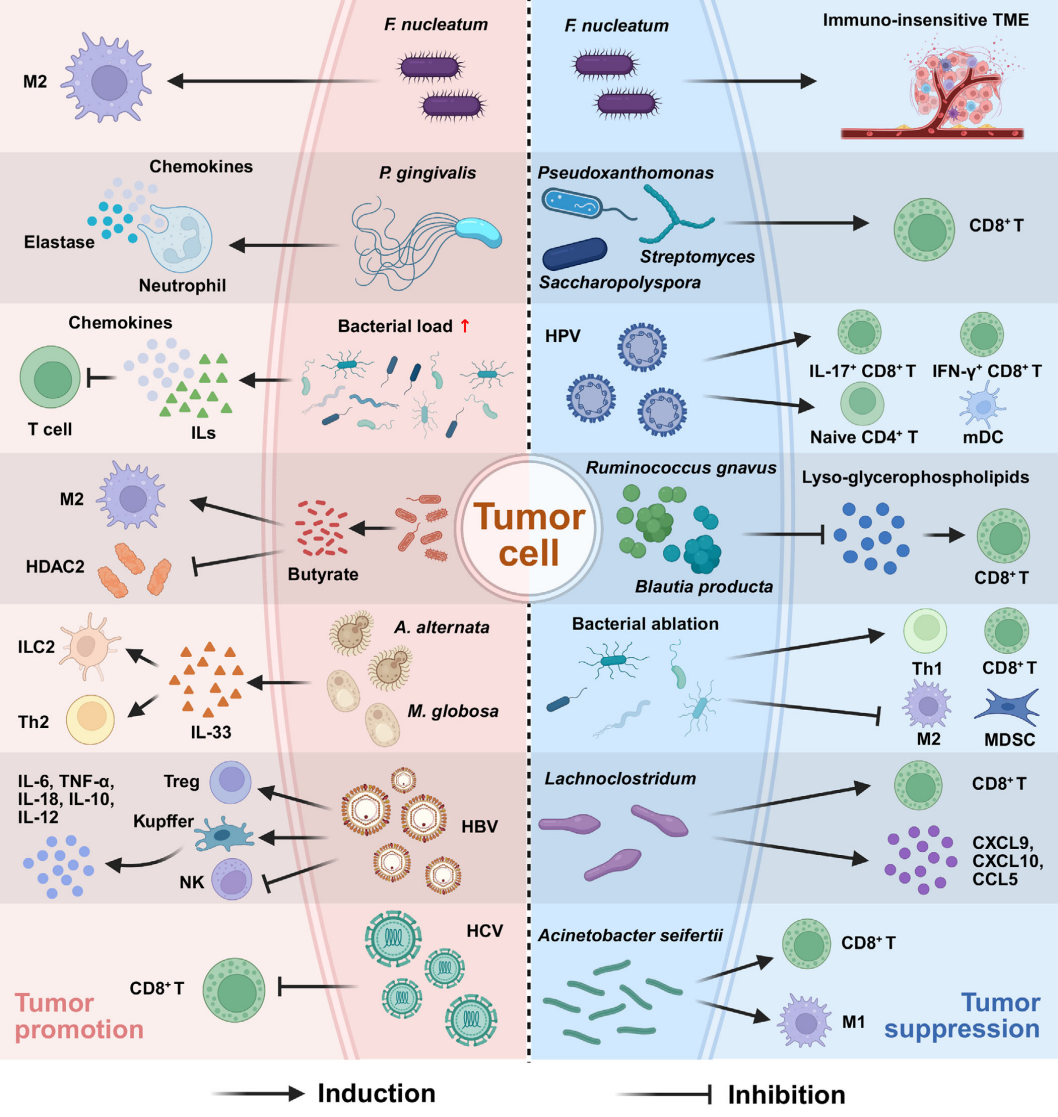
Dual roles of intratumoral microbiome in tumor development through multiple mechanisms regulating the immune microenvironment. The figure demonstrates how intratumoral bacteria, fungi, and viruses exert both protumorigenic and antitumorigenic effects by regulating immune cell activation and recruitment, modulating the secretion of inflammatory factors and chemokines, and comprehensively reshaping the tumor immune microenvironment. This figure was created using the tools provided by Biorender.com (accessed on 08/04/2025). HBV, hepatitis B virus; HCV, hepatitis C virus; HDAC2, histone deacetylase 2; HPV, human papillomavirus; IFN‐γ, interferon‐γ; IL, interleukin; ILC2, type 2 innate lymphoid cells; mDC, myeloid DC; MDSC, myeloid‐derived suppressor cell; M1, M1 macrophage; M2, M2 macrophage; NK, natural killer; Th1, T helper 1 cell; Th2, T helper 2 cells; TME, tumor microenvironment; TNF‐α, tumor necrosis factor‐α.

Intratumoral microbiome exerts dual effects on tumor development through diverse mechanisms that regulate the immune microenvironment (Figure 3). On one hand, certain microorganisms disrupt host immune homeostasis by inducing immunosuppression and promoting chronic inflammation, thereby creating a conducive microenvironment for tumor initiation and progression. On the other hand, specific microorganisms can activate immune cells and enhance antigen presentation processes, enabling the immune system to efficiently recognize and eliminate tumor cells, thereby exerting anticancer immune effects. Therefore, the intratumoral microbiome can serve both as a potential therapeutic target and as a key factor that facilitates immunotherapy; comprehensive investigation of its immune regulatory mechanisms and molecular pathways will provide novel directions for tumor immunotherapy strategies.

#### 3.2.1 Intratumoral Microbiome Influence the Immune Microenvironment to Promote Tumor Initiation and Progression

Intratumoral microbiome significantly influences the tumor immune microenvironment by regulating key signaling pathways, modifying epigenetic states, and modulating the release of various inflammatory factors. Extensive research indicates that bacteria colonizing tumors can significantly promote tumor growth, with the specific intratumoral microbiome inducing IL‐17 production, which subsequently promotes B cell recruitment and infiltration into tumor tissues, thereby triggering CRC progression. For instance, *Fusobacterium nucleatum* can promote M2 macrophage activation, directly participating in carcinogenesis, whereas *Porphyromonas gingivalis* can specifically stimulate neutrophils to secrete chemokines and elastase, thereby promoting tumor progression. In NPC, intratumoral bacterial load negatively correlates with T lymphocyte infiltration into tumor tissues, as these bacteria enhance the secretion of specific chemokines and various ILs in the surrounding microenvironment, causing T cell exclusion—a phenomenon suggesting that NPC microbiome can effectively suppress antitumor immunity [48]. Recent studies have demonstrated that the role of butyrate in tumors is concentration‐dependent, with low concentrations promoting LC cell proliferation, migration, and invasion, while high concentrations inhibiting LC cell proliferation [165]. This phenomenon can be attributed to the ability of low‐concentration butyrate to promote pathways related to cell adhesion and cell–cell junctions, while bacterially derived butyrate inhibits histone deacetylase 2 (HDAC2) and activates M2 macrophage polarization, subsequently inducing the expression of long noncoding RNA H19 in tumor cells, thereby facilitating LC metastasis. However, under certain conditions, macrophage depletion may occur, and this reduction in macrophages can significantly abrogate the metastasis‐promoting effects of butyrate [165]. Intratumoral fungi *Alternaria alternata* and *Malassezia globosa* can promote the release of IL‐33, which subsequently recruits type 2 innate lymphoid cells (ILC2) and T helper 2 cells (Th2) to the TME [166].

Intratumoral viruses likewise play crucial roles in carcinogenesis. Research has established that after HPV infects epithelial cells, its viral particles often remain confined to the uppermost layer of epithelial cells, which represents a blind spot in the host's immune detection mechanisms. This “geographical advantage” enables HPV to effectively evade immune surveillance, resulting in long‐term chronic HPV infection that significantly promotes cancer development [167]. In HBV‐induced carcinogenesis, HBV can stimulate Treg production and promote an increase in NK cell numbers accompanied by selective functional defects, thereby leading to an overall state of host immune deficiency. Furthermore, HBV can activate Kupffer cells in the liver to secrete IL‐6 and tumor necrosis factor (TNF)‐α, while simultaneously inducing the production of IL‐18, IL‐10, and IL‐12, which collectively promote the malignant transformation of hepatocytes [56, 168, 169]. Clinical and experimental studies indicate that chronic HCV infection causes functional exhaustion of CD8^+^ T cells through persistent antigenic stimulation and induces elevated expression of PD‐1, thereby weakening host immune surveillance capability and facilitating tumor immune escape [169, 170]. Additionally, HIV, as a recognized immunosuppressive factor, can induce host immune evasion and frequently acts synergistically with other pathogenic microorganisms, significantly contributing to the development of various malignancies including lymphoma [171].

#### 3.2.2 Intratumoral Microbiome Assist in Exerting Anticancer Immune Effects

Regarding tumor suppression, the intratumoral microbiome can inhibit tumor initiation and progression through various mechanisms, including regulating the TME, influencing gene mutations, and modulating the function of genetic material in tumor cells, thereby significantly affecting therapeutic responses and potentially serving as valuable targets for cancer treatment [172]. Current research indicates that *F. nucleatum* remodels the TME around oral squamous cell carcinoma tissue to be insensitive to immune responses, while *Pseudoxanthomonas*, *Saccharopolyspora*, and *Streptomyces* genera can stimulate CD8^+^ T cell infiltration, thereby promoting antitumor immunity [48]. In addition, HPV in head and neck squamous cell carcinoma (HNSCC) tumor tissues was also associated with the infiltration of a variety of immune cells, including IL‐17^+^ CD8^+^ T lymphocytes, IFN‐γ^+^ CD8^+^ T lymphocytes, naive CD4^+^ T lymphocytes, and myeloid DCs. HPV‐positive HNSCC patients thus exhibit a strong antitumor immune response and are associated with a favorable prognosis [173]. *Ruminococcus gnavus* and *Blautia producta* in colon tumors relieve the inhibitory effect on CD8^+^ T cells by degrading lyso‐glycerophospholipids, thereby promoting immune surveillance function and inhibiting the progression of CRC [174]. In pancreatic tumor‐related studies, bacterial ablation has been found to promote Th1 cell and CD8^+^ T cell infiltration into the TME while simultaneously inhibiting the activity of M2 tumor‐associated macrophages and immunosuppressive MDSCs. Notably, fecal microbiome suppression in tumor‐bearing KPC mice counteracts the beneficial effects of bacterial ablation [175]. In melanoma, a variety of intratumoral microorganisms (especially *Lachnoclostridum*) can increase the infiltration of CD8^+^ T cells in the immune microenvironment, and are positively correlated with the levels of chemokines CXCL9, CXCL10, and CCL5, which is helpful to improve immunotherapy and improve the survival rate of patients [176]. Studies have shown that *Acinetobacter seifertii* is significantly enriched in immunoenriched ovarian cancer patients, and the infiltration of CD8^+^ T cells and M1 macrophages in the immune microenvironment is increased. Compared with immunodeficient ovarian cancer, immunoenriched ovarian cancer patients show better survival [177].

### 3.3 Relationship Between Intratumoral Microbiome and ICI Efficacy

As previously mentioned, the intratumoral microbiome can influence or even reshape the tumor immune microenvironment, indicating that the intratumoral microbiome is closely related to the efficacy of ICIs, a hypothesis that has been validated by numerous studies. Research demonstrates that prostate‐specific microorganism CP1, when administered via transurethral injection, can specifically colonize prostate cancer tumor tissue, significantly enhancing antitumor immune responses, including promoting T cell activation, DC maturation, enhancing macrophage function, and increasing NK cell activity, while simultaneously promoting immune cell infiltration into tumor tissues and inhibiting Treg activation [178]. Moreover, CP1 exhibits significant synergistic effects with anti‐PD‐1 immunotherapy, not only inhibiting tumor cell proliferation but also improving patient survival rates [178]. Studies have demonstrated that bacteria within melanoma cells similarly influence ICI efficacy. Specifically, intratumoral bacteria inhibit cancer cell immune escape mechanisms by presenting bacterial peptides to MHC molecules, promoting T cell infiltration and enhancing their cytotoxic capacity against tumor cells, ultimately improving ICI efficacy [61]. Conversely, research indicates that intratumoral bacteria can release pathogen‐associated molecular patterns (PAMPs) such as flagellin and LPS, activating mitogen‐activated protein kinase (MAPK), Janus kinase‐signal transducer and activator of transcription (JAK‐STAT), and NF‐κB signaling pathways, thereby recruiting immunosuppressive MDSCs and neutrophils to the TME [131]. This process leads to upregulation of immune checkpoint molecules, CTLA‐4 and PD‐1, while simultaneously inhibiting CD4^+^ and CD8^+^ T cell infiltration into bacterial‐enriched regions, thereby establishing an immunosuppressive barrier. Research confirms that this tumor‐bacteria symbiotic relationship constitutes a key mechanism for immune evasion [131]. Clinical studies further demonstrate that the diversity of the intratumoral microbiome significantly impacts ICI efficacy, with patients achieving a high objective response rate (ORR) exhibiting notably higher microbial diversity than those with a low ORR. Additionally, bacteria of the *Eudoraea* and *Desulfonatronospira* genera show significantly increased abundance in the high ORR group [179]. Notably, treatment regimens combining anti‐PD‐1 with *Eudoraea* can activate CD8^+^ T cell and NKT cell functions, significantly enhancing the efficacy of immunotherapy in mouse tumor models [179]. Additionally, research indicates that *Bifidobacterium* in the intestine can translocate to tumor tissues under high‐salt dietary conditions, subsequently enhancing NK cell function, ultimately promoting tumor regression [180]. Collectively, these studies suggest that the abundance of specific bacterial genera within tumors and the overall diversity of microbial communities have considerable potential to serve as important biomarkers for predicting and improving ICI efficacy by regulating T cell activity and reshaping the immunosuppressive microenvironment. Multiple studies confirm that intratumoral injection of *Bifidobacteria* can activate NK cells and effectively inhibit tumor growth, and is therefore considered a potential adjunctive strategy to enhance immunotherapy efficacy [180]. In animal models, the experimental results demonstrated that *B. fragilis* administered orally could colonize the gastrointestinal mucosal layer of germ‐free mice. Subsequently, it induced an antitumor immune microenvironment, promoted the Th1 type helper T‐cell immune response, and the maturation of DCs within the tumor, thereby significantly enhancing the treatment response of mice to the CTLA‐4 antibody [122]. Beyond traditional microbial therapies, researchers are also developing novel treatment strategies by combining microorganisms with nanomaterials. For example, complexes of *F. nucleatum*‐specific bacteriophages and silver nanoparticles have been demonstrated to reduce the abundance of tumor‐promoting microbiome and inhibit tumor cell proliferation, thereby enhancing the efficacy of ICIs [181].

Overall, the intratumoral microbiome bidirectionally regulates the tumor immune microenvironment through multiple mechanisms, potentially both promoting immune evasion and enhancing antitumor immune responses. Their compositional characteristics are closely correlated with immunotherapy efficacy, demonstrating potential as predictive biomarkers or therapeutic targets. Future research necessitates further multiomics and functional studies to elucidate their specific mechanisms of action and facilitate clinical translation.

## 4 Microbiome From Other Sources and Cancer Immunotherapy

Beyond the gut, microbial communities from multiple ecological niches, including the oral cavity, skin, respiratory tract, and urogenital tract, also play important roles in regulating cancer immunotherapy (Table 2). Oral microbiome primarily influences immune responses in head and neck, gastrointestinal, and respiratory tract tumors through dynamic changes in specific bacterial composition and abundance, as well as by inducing local inflammatory responses, while the skin microbiome not only participates in shaping systemic immune status but also directly regulates the immune characteristics of skin‐related TME. Consequently, skin microbiome‐based intervention strategies have demonstrated significant antitumor effects in multiple studies. The composition and function of the respiratory tract microbiome significantly affect LC patients’ responsiveness to immunotherapy and clinical outcomes, while the urogenital tract microbiome primarily influences immunotherapy responses in urogenital system tumors by regulating the balance of proinflammatory and anti‐inflammatory factors and activating specific signaling pathways. In summary, these diverse microbial communities participate in the regulation of cancer immunotherapy through complex and specific mechanistic networks, providing important theoretical foundations and potential therapeutic targets for developing cross‐niche, multitarget microbiome intervention strategies.

**TABLE 2:** Regulatory influence of microbial communities from the oral cavity, skin, respiratory system, and urogenital tract on cancer immunotherapeutic efficacy.

Organ system	Microorganism	Cancer types	Model	Immunotherapy	Mechanism	Clinical significance	References
**Oral cavity**	HPV	HNC	Human	Anti‐PD‐1 immunotherapy	Reconstitution of CD8^+^ T cell function.	Enhanced antitumor immune responses	[182, 183]
	*N. subflava* *N. perflava* *N. flavescens* *Actinomyces meyeri* *A. hongkongensis* *A. georgiae*	NSCLC	Human	Anti‐PD‐1 immunotherapy	Stimulate macrophage secretion of IL‐6‐related signaling pathways; Activate human monocyte‐derived DCs.	Immunotherapy had a positive response	[184]
	*Granulicatella adiacens* *Streptococcus oralis*	NSCLC	Human	Anti‐PD‐1 immunotherapy	The production of IL‐1β, IL‐8, and TNF‐α by peripheral blood mononuclear cells was induced Upregulated; PI3K signaling pathway.	Immunotherapy had a poor response	
	*Peptostreptococcus anaerobius*	CRC	Mouse	Anti‐PD‐1 immunotherapy	Promote the recruitment and activation of MDSCs.	Promoting drug resistance	[185]
**Skin**	*Bifidobacterium longum* *Collinsella aerofaciens* *Enterococcus faecium*	Metastatic melanoma	Mouse	Anti‐PD‐1 immunotherapy	Reduced Tregs; Batf3 DC increased Enhanced Th1 responses.	Improve the efficacy of anti‐PD‐L1 therapy	[115]
**Respiratory**	*Veillonella parvula*	Lung cancer	Mouse	Anti‐PD‐1 immunotherapy	IL‐17, PI3K, MAPK, and ERK pathways were upregulated; Activation of the IL‐17 inflammatory factor; Increased expression of immune checkpoint‐related markers.	Inhibition of anti‐PD‐1 immune response	[186]
	*Pseudomonas aeruginosa*	Lung cancer	Mouse	Anti‐PD‐1 immunotherapy	Induction of chronic inflammation; Formation of an immunosuppressive microenvironment.	Inhibition of anti‐PD‐1 immune response	[187]
	*B.thetaiotaomicron* *B. fragilis*	Melanoma CRC	Human	Anti‐CTLA‐4 immunotherapy	Induction of specific T‐cell immune responses.	The antitumor response to CTLA‐4 Ab was restored	[122]
**Urogenital**	*Lactobacillus*	Carcinoma of urinary bladder	Human	BCG immunotherapy	Induction of antiproliferative and cytotoxic effects.	Enhance the efficacy of BCG	[188]
	HPV	Cervical cancer HNSCC	Human	ICB	Regulates the number of TLS; Impact on TME.	Reduce the efficacy of ICB	[189]

Abbreviations: BCG, Bacillus Calmette–Guerin; CRC, colorectal cancer; DCs, dendritic cells; ERK, extracellular signal‐regulated kinase; HNC, head and neck cancer; HPV, human papillomavirus; IL, interleukin; MAPK, mitogen‐activated protein kinase; MDSCs, myeloid‐derived suppressor cells; NSCLC, non–small‐cell lung cancer; PI3K, phosphatidylinositol 3‐kinase; TLS, tertiary lymphoid structures; TME, tumor microenvironment; TNF, tumor necrosis factor; Tregs, regulatory T cells.

### 4.1 Oral Microbiome and Cancer Immunotherapy

The oral microbiome consists of various microorganisms, including *F. nucleatum*, *Neisseria*, *Candida albicans*, and HPV, with certain microbial communities being closely associated with cancer immunotherapy efficacy. Research indicates that oropharyngeal dysbiosis may significantly impact head and neck cancer treatment outcomes and is closely associated with adverse reactions to radiotherapy, chemotherapy, and immunotherapy [190]. For head and neck cancer patients, HPV‐positive status is associated with immune response mechanisms to PD‐1 blockade, with anti‐PD‐1/PD‐L1 treatment potentially serving as an effective therapeutic strategy by restoring CD8^+^ T cell function to enhance antitumor immune responses. These findings suggest that HPV status may influence the efficacy of anti‐PD‐1/PD‐L1 therapy [182, 183]. Current research findings suggest that targeted modulation of the oral microbiome can significantly improve cancer patients’ responses to immunotherapy. Metabolic disorders induced by salivary microbial imbalance can affect CD8^+^ T cell activity and PD‐L1 expression levels, a phenomenon closely associated with differential responses and resistance to immunotherapy in NSCLC patients, thereby providing a basis for developing personalized clinical immunotherapy regimens. In NSCLC patients receiving immunotherapy with favorable treatment responses, the relative abundance of *Neisseria* species, such as *N. subflava*, *N. perflava*, and *N. flavescens*, is significantly increased, suggesting these microorganisms may serve as potential biomarkers for predicting clinical prognosis and enhancing the efficacy of immunotherapy. Among these, *N. subflava* enrichment is most common and significantly associated with longer PFS in patients [184]. Research has found that *Actinomyces meyeri*, *A. hongkongensis*, and *A. georgiae* positively correlate with favorable immunotherapy responses and PD‐L1 expression levels, while *Granulicatella adiacens* and *S. oralis* negatively correlate with immunotherapy efficacy, with *S. oralis* specifically confirmed to impede clinical outcomes of immunotherapy [184]. Studies in CRC mouse models have found that *Peptostreptococcus anaerobius* can promote the recruitment of MDSCs and enhance their immunosuppressive activity, thereby eliminating the therapeutic effects of anti‐PD‐1 treatment [185]. Notably, immunotherapy itself can influence the community composition and relative abundance of the oral microbiome. Experimental studies demonstrate that in mouse models treated with anti‐CD47 monoclonal antibodies, the α‐diversity of the oral microbiome significantly decreases, with markedly reduced abundances of *Staphylococcus*, *Jeotgalicoccus*, and *Sporosarcina* [191].

### 4.2 Skin Microbiome and Cancer Immunotherapy

The skin tissue harbors a rich and diverse microbial community; in recent years, concurrent with the widespread application of immunotherapy in skin cancers, an increasing number of studies have demonstrated that the skin microbiome can significantly influence the clinical efficacy of cancer immunotherapy. Research has demonstrated that in melanoma patients receiving ICIs, the composition of the skin microbiome significantly correlates with both immunotherapy efficacy and tolerability. Notably, when ATBs disrupt patients’ microbial balance, those receiving ICI therapy often exhibit markedly poorer clinical outcomes [64]. Furthermore, melanoma‐related studies have revealed that OV therapy possesses dual immunomodulatory effects: inducing T cell infiltration into the TME while simultaneously inhibiting PD‐L1‐mediated negative immune regulation. This synergistic mechanism demonstrates considerable potential in overcoming tumor resistance to ICIs caused by high PD‐L1 expression, thereby significantly enhancing ICI therapeutic efficacy [192]. Cutting‐edge animal model research demonstrates that genetically engineered *Staphylococcus epidermidis*, following colonization of mouse skin, can specifically express melanoma‐associated antigens, thereby enhancing T cell‐mediated antitumor immune responses. This innovative microbial intervention strategy provides a novel adjuvant therapeutic approach for optimizing melanoma immunotherapy [193]. In summary, the skin microbiome plays a critical role in regulating both the efficacy and resistance mechanisms of immunotherapy for skin tumors. These research findings suggest that through precise regulation or genetic engineering of the skin microbiome, when combined with existing immunotherapy strategies, more effective comprehensive cancer treatment approaches may be developed, thus providing new research directions for personalized precision cancer therapy.

### 4.3 Respiratory Tract Microbiome and Cancer Immunotherapy

The respiratory tract microbiome is closely associated with the development of various respiratory diseases, including acute respiratory infections, asthma, chronic obstructive pulmonary disease, and LC, while concurrently significantly impacting the efficacy of cancer immunotherapy for LC. Research has established that dysbiosis of the lung microbiome can promote lung carcinogenesis by modulating the host immune system and reshaping the local immune microenvironment, a process primarily involving molecular mechanisms such as activation of TLR signaling pathways and release of TNF, which similarly participate in regulating cancer immunotherapy processes [194]. In LC mouse models, the lower respiratory tract microbe *Veillonella parvula* is significantly associated with upregulation of IL‐17, phosphatidylinositol 3‐kinase (PI3K), MAPK, and extracellular signal‐regulated kinase (ERK) pathways, thereby activating IL‐17 inflammatory factors and increasing immune checkpoint‐related marker expression. Importantly, these microbial community alterations are closely linked to poor patient prognosis and directly affect the efficacy of ICI therapy [186]. Studies demonstrate that the oral microbiome can reach the respiratory tract through inhalation or hematogenous spread, subsequently promoting LC progression through multiple mechanisms, including chronic inflammation induction, immune microenvironment reprogramming, and activation of carcinogenic signaling pathways. Consequently, detection and intervention of specific microbes hold promise as biomarkers for predicting cancer immunotherapy responses and as potential targets for enhancing immunotherapy efficacy [195]. LPS from *Pseudomonas aeruginosa* can induce chronic inflammation and facilitate the formation of an immunosuppressive microenvironment, with research having confirmed that these alterations are closely associated with the efficacy of PD‐1 inhibitor therapy in mice [187]. Additionally, members of the *Bacteroides* genus present in the lower respiratory tract, specifically *B. thetaiotaomicron* and *B. fragilis*, can significantly influence the therapeutic efficacy of ipilimumab (a CTLA‐4 inhibitor) by inducing specific T‐cell immune responses [122]. In summary, the composition and dynamic changes of the respiratory tract microbiome are closely related to the regulation of local inflammatory responses and the immune microenvironment, exerting critical impacts on the efficacy of tumor ICI therapy through multiple mechanisms, including the modulation of both innate and adaptive immune responses.

### 4.4 Urogenital Tract Microbiome and Cancer Immunotherapy

The human urinary and reproductive systems are colonized by diverse microbial communities that not only are closely associated with the pathophysiological processes of urogenital system diseases but also significantly impact the clinical efficacy of cancer immunotherapy. Research indicates that specific intratumoral microbes, such as *Listeria monocytogenes*, *Methylobacterium radiotolerans JCM 2831*, and *Xanthomonas albilineans GPE PC73*, can inhibit tumor cell proliferation by promoting immune cell infiltration into the TME [196]. Studies show that the postoperative recurrence risk in bladder cancer patients receiving transurethral resection of bladder tumor and intravesical BCG immunotherapy is closely related to their baseline urinary microbiome composition; therefore, systematic analysis of changes in urinary microbiome characteristics before and after treatment holds promise for developing microbial markers to predict treatment response [188]. In cervical cancer and HNSCC, HPV infection status exhibits a significant correlation with the number of tertiary lymphoid structures (TLS), which, as key components of the TME, can effectively inhibit tumor growth and are closely associated with improved prognosis in cancer patients [189]. Studies proposed that the urinary microbiome is a potential key factor influencing the development and progression of breast cancer, with these microorganisms participating in disease processes by modulating local bladder immune responses [197]; consequently, precise regulation of urinary system microbial communities may represent an innovative strategy for the prevention and treatment of bladder cancer. Although the association between the urinary microbiome and urological malignancies has gained preliminary recognition, systematic exploration of the molecular mechanisms linking the urogenital tract microbiome with cancer immunotherapy efficacy remains limited, thus necessitating in‐depth research to reveal their intrinsic connections and provide new theoretical foundations and potential targets for optimizing immunotherapy strategies for urogenital system tumors.

In summary, microbial communities from oral, skin, respiratory, and urogenital sites significantly influence the efficacy of tumor immunotherapy by modulating local immune microenvironments, inflammatory responses, and cytokine networks. These findings expand the scope of microbiome research and suggest that coordinated regulation by multiniche microbial communities may represent a novel strategy for future tumor immunotherapy.

## 5 Application Prospects of Microbiome Research in Cancer Immunotherapy

**FIGURE 4:**
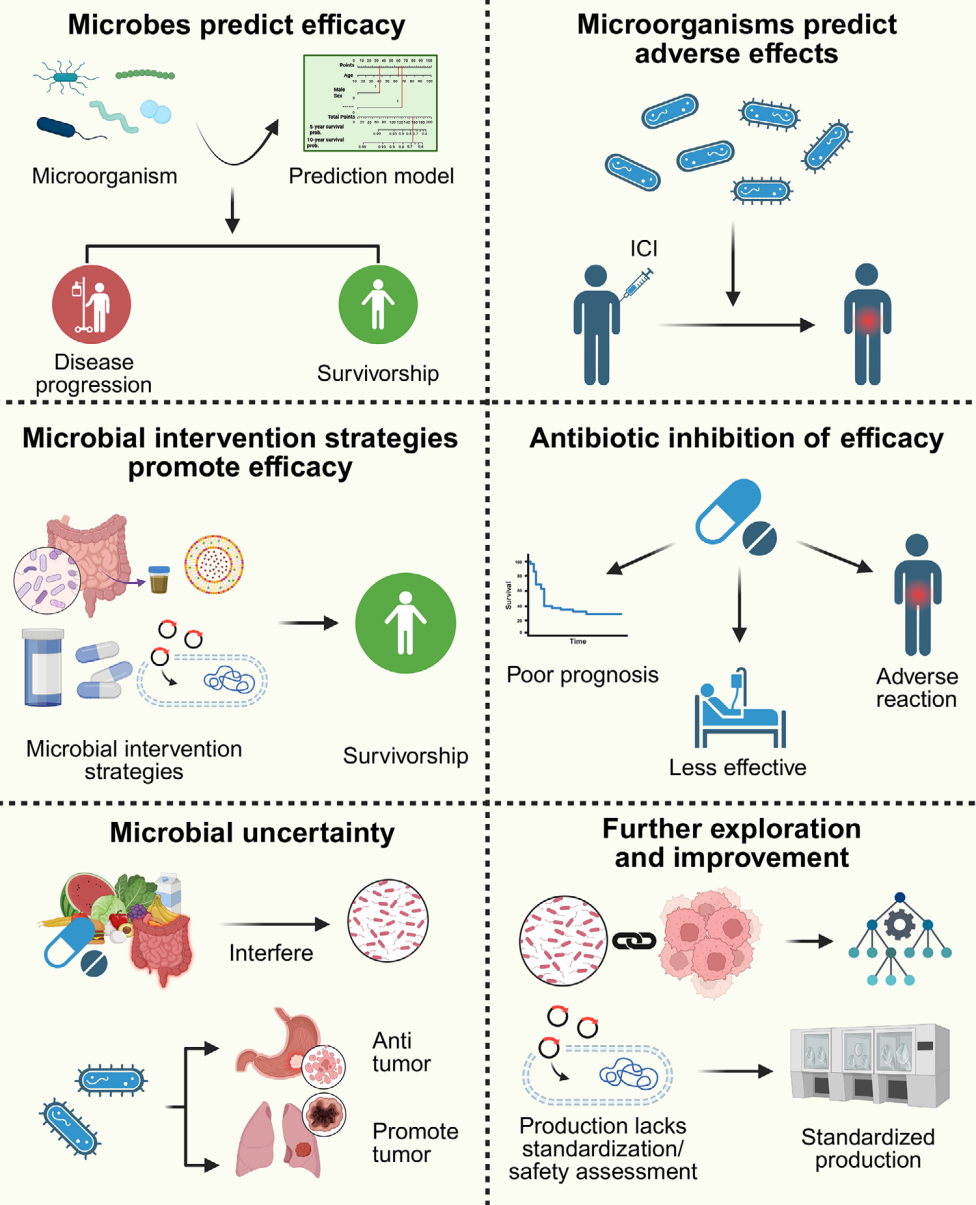
Prospects for microbiome research applications in cancer immunotherapy. The microbiome can be utilized to predict ICI therapy efficacy and adverse event rates; additionally, microbiome‐targeted intervention strategies can enhance ICI therapeutic effects, whereas ATBs frequently suppress treatment efficacy. The dynamic and variable nature of the microbiome poses significant challenges for microbiome research in cancer immunotherapy, necessitating more comprehensive exploration. This figure was created using the tools provided by Biorender.com (accessed on 08/04/2025). ICI, immune checkpoint inhibitor.

Microbiome research has established innovative pathways for cancer immunotherapy and demonstrated tremendous application potential (Figure 4). By analyzing specific microbiome characteristics, researchers can develop predictive models for immunotherapy efficacy, thereby providing critical biomarker support for individualized treatment decisions. Microbiome‐based manipulation strategies, such as probiotic interventions, microbial transplantation, and regulation of microbial metabolites, can reshape the tumor immune microenvironment, potentially overcoming tumor resistance to immunotherapy. Microbiome research is driving cancer immunotherapy toward precision medicine, thereby facilitating the development of personalized immunotherapy protocols to address individual variations in treatment efficacy. Despite numerous challenges facing microbiome research in cancer immunotherapy, its significant association with immunotherapy responses strongly suggests that this field will become a critical direction for optimizing cancer immunotherapy and will provide novel opportunities for improving patient prognosis.

### 5.1 Microbiome as a Biomarker for Predicting Cancer Immunotherapy Efficacy

As previously mentioned, characteristic changes in the microbiome are closely associated with immunotherapy efficacy for multiple tumor sites; therefore, specific microbiome features are considered potential biomarkers for predicting immunotherapy efficacy. Research indicates that gut microbiome diversity in patients receiving anti‐PD‐1 therapy is significantly positively correlated with treatment response [114, 198]. Derosa et al. [199] demonstrated that *Dorea longicatena*, *Dorea formicigenerans*, *E. rectale*, and *Streptococcus salivarius* were significantly enriched in healthy patients and in patients with RCC who had not received ATB treatment, while *Clostridium ramosum* and *Alistipes senegalensis* were enriched in patients who responded to immunotherapy without ATB use. This study indicates that differences in microbial communities are closely related to varying responses to ICI therapy, thereby highlighting the potential of the microbiome as a biomarker for predicting immunotherapy efficacy. In patients with metastatic melanoma, those receiving ipilimumab immunotherapy may experience colitis as an adverse reaction, which is significantly positively correlated with *Firmicutes* enrichment and significantly negatively correlated with *Bacteroidetes* [136]. Through univariate Cox proportional hazards analysis, *Faecalibacterium* and *Bacteroides* were identified as promising predictive indicators [114]. Zhu et al. established a model based on 18 bacterial species by analyzing metagenomics, internal transcribed spacer 2 (ITS2), and metabolomics data to predict whether advanced HCC patients receiving ICI therapy would derive sustained benefit, and identified biomarkers for predicting patient survival rates after immunotherapy, including two bacteria (*Senegalimassilia_anaerobia* and *Actinomyces_sp_ICM47*) and one metabolite (galanthaminone) [200]. Another study also indicated that the *Bacteroidetes phylum* and the *Clostridium phylum* could predict patient responsiveness to anti‐PD‐1 therapy [201]. *Akkermansia* species, the *Firmicutes*/*Bacteroidetes* ratio, and the *Prevotella*/*Bacteroides* ratio can serve as biomarkers for predicting the efficacy of monoclonal antibody nivolumab therapy, with *Akkermansia* significantly associated with groups of patients who respond well to treatment [62]. Additionally, microbial metabolites are also closely related to cancer immunotherapy outcomes. Research indicates that butyrate produced by gut microbial metabolism is positively correlated with the efficacy of anti‐PD‐1 immunotherapy and can serve as an effective biomarker for predicting treatment response [202]. Significant increases in bacterial metabolite concentrations were also observed in patients receiving CAR‐T therapy, with these metabolites being considered potential biomarkers for evaluating treatment efficacy [203].

The intratumoral microbiome is intimately associated with immunotherapy efficacy and patient prognosis, demonstrating promise as a potential biomarker for predicting immunotherapy outcomes. Research indicates that the abundance of *Methylobacterium* in tumor tissues of GC patients significantly correlates with poor prognosis, likely operating by reducing the proportion of CD8^+^ tissue‐resident memory T (TRM) cells in the TME and downregulating TGF‐β expression, thereby suppressing antitumor immune responses [204]. This discovery has revealed the potential value of *Methylobacterium* as a prognostic biomarker. In CRC, colibactin‐producing *Escherichia coli* (CoPEC) can induce an immune microenvironment with protumor characteristics, leading to reduced antitumor T cell counts and subsequently triggering resistance to anti‐PD‐1 immunotherapy in tumor tissues; consequently, CoPEC is considered a potential microbial biomarker for predicting immunotherapy efficacy [205]. Conversely, long‐term survivors of pancreatic ductal adenocarcinoma exhibit significantly increased microbiome α‐diversity, with specific tumor microbiome members (*Pseudoxanthomonas*, *Streptomyces*, *Saccharopolyspora*, and *Bacillus clausii*) orchestrating favorable antitumor immune microenvironments by promoting CD8^+^ T cell activation and infiltration into tumor tissues, further confirming the value of intratumoral microbial communities as potential biomarkers for predicting immunotherapy prognosis [54]. Jang et al. [206] analyzed the bronchial microbiome composition of NSCLC patients, revealing that the relative abundance of *Firmicutes*, *Bacteroidetes*, and *Veillonella* was significantly higher in high PD‐L1 expression and immunotherapy‐responsive groups compared to low PD‐L1 expression and nonresponsive groups, with Veillonella being particularly predominant in both high PD‐L1 expression and immunotherapy‐responsive patient populations, suggesting these specific bacterial taxa may serve as novel biomarkers for predicting anti‐PD‐L1 immunotherapy efficacy. Trimethylamine N‐oxide (TMAO) is a well‐established metabolite produced by the metabolic activity of the *Clostridiales* genera. In TNBC patients, significantly elevated concentrations of TMAO were observed in tumor tissues situated within immunologically active microenvironments, with this metabolite demonstrating the capacity to induce tumor cell pyroptosis and enhance CD8^+^ T cell‐mediated antitumor immune responses, thereby significantly improving patient responses to anti‐PD‐1 immunotherapy [207]. Recent research demonstrates that the probiotic *Limosilactobacillus reuteri* colonizing melanoma tissues can release the tryptophan metabolite indole‐3‐acetic acid (I3A), which potently activates CD8^+^ T cells and significantly enhances the therapeutic efficacy of ICIs [208]. Additionally, experiments with *Bifidobacterium* combined with anti‐CD47 immunotherapy in tumor‐bearing mouse models demonstrate that systemically administered *Bifidobacterium* can selectively accumulate in the TME, while local administration can trigger activation of the stimulator of interferon genes (STING) pathway, synergistically enhancing clinical responses to anti‐CD47 immunotherapy [209].

In recent years, targeted therapeutic strategies against the intratumoral microbiome have increasingly demonstrated clinical translational potential. For instance, interventions targeting *F. nucleatum* have been shown to modulate the immune microenvironment in CRC, thereby enhancing the efficacy of PD‐1 inhibitors [210]. The engineered *Escherichia coli* Nissle 1917 (EcN) vector platform can target tumor‐derived exosomes (TDEs) to reverse tumor immune suppression, demonstrating potential for enhancing immunotherapeutic responses in preclinical studies [211]. These studies have established the groundwork for developing novel microbiome‐based combination immunotherapy regimens [131, 212]. However, most microbial intervention strategies remain in preclinical or early clinical phases, with their efficacy and safety requiring further validation. Nevertheless, targeting the intratumoral microbiome represents a promising approach for developing new therapeutic directions that may enhance immunotherapeutic efficacy and improve patient outcomes. Currently, numerous completed and ongoing clinical studies have investigated the impact of microbiome intervention strategies on tumor immunotherapy (Table 3). These studies are expected to provide valuable insights into the many challenges that currently exist in microbiome interventions (primarily FMT) and tumor immunotherapy.

**TABLE 3:** Clinical trial research exploring the association between microbial intervention strategies and tumor immunotherapy.^a^

Study	Study summary	Tumor	Interventions	Endpoint/result	Status	NCT
Phase II, 48	To evaluate the safety and immunogenicity of FMT combined with standard immunotherapy in patients with advanced HCC.	HCC	FMT, Vancomycin oral capsule, ATB, immunotherapy	Not yet available	Recruiting	NCT05690048
Phase I, 10	To determine whether FMT capsules can help reverse the drug resistance of patients with gastrointestinal cancer to PD‐(L)1 therapy.	Gastrointestinal system cancer	FMT capsule	Not yet available	Completed	NCT04130763
Phase I, Phase II, 50	To evaluate the improvement effect of FMT performed on donors who responded to ICIs on the response rate of patients with advanced RCC to ICI therapy.	RCC	donor FMT, Placebo FMT	Not yet available	Active, not recruiting	NCT04758507
Phase II, 62	By combining FMT with standard first‐line treatment methods, the antitumor immune effect is enhanced, the PFS period of patients is prolonged, and the prognosis of patients is improved.	NSCLC	Combination product: FMT + chemotherapy + immunotherapy	The primary endpoint of this study is the 12‐month PFS rate.	Recruiting	NCT06403111
Phase I, 20	To investigate whether FMT performed before the initiation of immunotherapy combination therapy can prevent the toxic reactions caused by the combination therapy of ipilimumab and nivolumab.	RCC	FMT	FMT is beneficial for dealing with subsequent microbial challenges caused by immune suppression in cancer.	Active, not recruiting	NCT04163289
Phase II, 128	To investigate whether it is possible to reduce the chances of melanoma growth or spread, when receiving the FMT treatment (as a supplement to the conventional immunotherapy ICB treatment)	Melanoma	Standard of care ICB, LND101	Combining first‐line PD‐1 therapy with oral FMT is safe and shows improvements in ORR, mPFS and mOS.	Recruiting	NCT06623461
Phase IV, 20	To evaluate the efficacy and safety of FMT combined with concurrent chemoradiotherapy and the ICI xindilimab as neoadjuvant therapy for locally advanced rectal cancer.	Localized advanced rectal adenocarcinoma	Immunotherapy, FMT capsules, Standard long course radiotherapy, Surgical resection of the mesorectum	Not yet available	Enrolling by invitation	NCT06931808
Phase II, Phase III, 65	Evaluate the safety, tolerability and implantation efficacy when MET‐4 series drugs are used in combination with ICIs.	All solid tumors	MET‐4	Not yet available	Active, not recruiting	NCT03686202
Phase I, Phase II, 12	Explore the safe dosage of the gene transfer experiment, and observe whether these specialized antitumor cells (anti‐HPV E6 cells) can reduce HPV‐related tumors, and test the toxicity of this treatment method.	Vaginal cancer, Cervical cancer, Anal cancer, Penile cancer, Oropharyngeal cancer	Anti‐HPV E6 cells	The endpoint indicators include safety, objective tumor response rate, and duration of response. Explore the immunological associations related to the E6 TCR T cell therapy.	Completed	NCT02280811

Abbreviations: FMT, fecal microbiome transplantation; HCC, hepatocellular carcinoma; ICB, immune checkpoint blockade; ICI, immune checkpoint inhibitor; MAIT, mucosa‐associated invariant T; MET‐4, feasibility study of microbial ecosystem therapeutics; mOS, median overall survival; mPFS, median progression‐free survival; NSCLC, non–small‐cell lung cancer; ORR, objective response rate; PFS, progression‐free survival; RCC, renal cell carcinoma.

^a^ Data sources – clinical trial registry website: https://clinicaltrials.gov/.

However, the translation of the intratumoral microbiome into clinically applicable biomarkers continues to face significant methodological challenges. These include standardization of sample collection and processing (such as avoiding oral or environmental microbial contamination), harmonization of sequencing depth and technologies, standardization of bioinformatics analysis pipelines, and validation of research reproducibility across platforms and cohorts. Furthermore, the high interindividual heterogeneity and dynamic nature of microbial communities necessitate the establishment of stringent quality control standards and correction models within large‐scale, multicenter prospective clinical studies to ultimately achieve their translation from basic research to clinical predictive tools.

### 5.2 Applications of Microbiome Manipulation Strategies in Cancer Immunotherapy

Microbiome manipulation strategies are increasingly becoming an integral component of cancer immunotherapy, primarily encompassing FMT, oral probiotics and prebiotics, targeted microbial therapies, and engineered bacterial applications [89]. Among these approaches, microbial intervention strategies such as FMT, probiotics, and prebiotics have demonstrated significant potential for application in various cancer treatments. Notably, ATB use may disrupt the symbiotic balance between host and microbiome, thereby weakening the body's capacity to respond to immunotherapy, a phenomenon that is closely associated with poor outcomes in cancer immunotherapeutic approaches. On the other hand, engineered bacteria as innovative microbial intervention tools, while possessing broad developmental prospects, still face multifaceted technical and safety challenges that must be addressed during their clinical translation process. In summary, these microbiome manipulation strategies collectively establish a complex regulatory network of microbiome–immunity–tumor interactions, thereby providing new research directions and clinical intervention approaches for optimizing cancer immunotherapy.

#### 5.2.1 FMT for Cancer Treatment

FMT has been implemented in therapeutic strategies for cancer patients [213]. Studies have demonstrated that transplanting fecal material from patients who respond well to anti‐PD‐1 therapy into germ‐free mice results in positive immunotherapy responses in the recipient mice; conversely, fecal transplants from nonresponders to PD‐1 therapy fail to induce these therapeutic effects [214]. Similarly, clinical studies have found that fecal transplantation from donors who respond favorably to ICIs significantly improves treatment responsiveness in melanoma and RCC patients, with this enhanced efficacy closely associated with specific fecal microbiome compositions [65]. Multiple animal studies have demonstrated that fecal transplantation enriched with *Ruminococcaceae* and *Faecalibacterium*, or oral administration of *Bifidobacterium*, *Bacteroides*, or *Burkholderia*, can act synergistically with anti‐PD‐L1 or anti‐CTLA‐4 therapies, significantly enhancing the antitumor effects of immunotherapy [198]. However, FMT currently lacks comprehensive long‐term safety assessment data, and studies have reported potential adverse events, thus necessitating further systematic research and the development of standardized operational protocols for FMT's clinical translation in cancer immunotherapy [215].

In mechanistic studies exploring the combined application of microbial intervention strategies with ICIs, Huang et al. [216] employed multiomics analytical approaches to systematically evaluate the synergistic antitumor effects and underlying molecular mechanisms of FMT combined with anti‐PD‐1 therapy in CRC‐bearing mouse models. Results demonstrated that the combination therapy group exhibited significantly greater tumor suppression effects compared to either FMT alone or anti‐PD‐1 monotherapy groups, with statistically significantly higher long‐term survival rates. Specifically, microbiome analysis revealed significant enrichment of certain *Bacteroides* genus, including *B. thetaiotaomicron* and *B. fragilis*, in the intestines of mice receiving combination therapy, while *B. ovatus* was notably reduced; these characteristic alterations in gut microbiome composition may represent the key microbiological foundation that promotes the enhanced antitumor efficacy of anti‐PD‐1 immunotherapy [216]. Following FMT intervention in melanoma patients with poor response to anti‐PD‐1 therapy, studies identified significant changes in their immune microenvironment, primarily manifested as upregulated activation levels of tumor‐infiltrating CD8^+^ T cells, thereby partially counteracting the immunosuppressive state within the TME. Mechanistic studies indicate that FMT promotes remodeling of the host gut microbiome, transforming its composition into advantageous bacterial community structures that favor anti‐PD‐1 efficacy, thereby significantly improving these patients’ clinical responses to anti‐PD‐1 immunotherapy. However, some anti‐PD‐1 therapy‐refractory patients may remain unresponsive to FMT intervention strategies due to various factors, including host intrinsic immune deficiencies, FMT donor microbiome lacking key functional microbes, or failure of transplanted microbiome to successfully colonize the recipient's intestines [126]. Furthermore, for microsatellite‐stable metastatic CRC patients, although immunotherapy combined with antiangiogenic treatment has emerged as an important therapeutic strategy, this combination regimen can only partially improve but not completely overcome the clinical challenge of low response rates to immunotherapy in these patients. Recent clinical studies indicate that integrating FMT intervention with immunotherapy and antiangiogenic combination regimens to form a triplet therapeutic strategy demonstrates significantly improved clinical benefits and survival advantages in refractory microsatellite‐stable metastatic CRC patients, thus offering a highly promising novel treatment approach for this patient population that traditionally exhibits limited immunotherapy efficacy [217].

#### 5.2.2 Probiotics and Prebiotics Assisting Cancer Immunotherapy

Research has conclusively demonstrated that supplementation with probiotics and prebiotics enhances the efficacy of immunotherapy in cancer patients, promoting antitumor effects as part of synergistic treatment strategies. In mice experiencing colitis‐related adverse reactions following anti‐CTLA‐4 treatment, supplementation with *Bifidobacterium* significantly ameliorates colitis symptoms in mice with ATB‐disrupted gut microbiome [218]. In patients receiving ICI therapy, microbial metabolites (specifically inosine) effectively activate intestinal T cells, thereby enhancing immunotherapy efficacy [219]. Experimental studies have demonstrated that oral administration of *Bifidobacterium* combined with anti‐PD‐L1 therapy can almost completely inhibit melanoma cell growth [220]. In both melanoma and CRC‐bearing mouse models, combined treatment with IL‐2 and the beneficial commensal microbe *AKK* demonstrates significant antitumor efficacy. Specifically, *AKK* enhances IL‐2 efficacy by activating the TLR2 signaling pathway through Amuc protein while simultaneously maintaining intestinal barrier homeostasis. Consequently, IL‐2 combined with *AKK* is regarded as a promising novel combination therapeutic approach [221]. In melanoma mouse models, *Lactobacillus rhamnosus* treatment significantly enhances immunity against B16 melanoma lung metastasis, suggesting that probiotics could serve as effective adjunctive therapeutic approaches for inhibiting cancer metastasis [222].

In the TME, *Lactobacillus* can promote the activation of multiple immune cells, including macrophages, DCs, T cells, and NK cells, while concurrently stimulating immune factor production, thereby enhancing antitumor effects. Additionally, unmethylated dinucleotide repeat sequences in *Lactobacillus* DNA can promote activation of innate immune responses, which partially explains why *Lactobacillus* are widely applied in cancer treatment and prevention [223]. A recent study comprehensively analyzed the impact of three next‐generation probiotics (NGPs) on cancer incidence, revealing that these probiotics may function through the following mechanisms: (1) enhancing gastrointestinal immune environments and maintaining intestinal barrier integrity, (2) secreting beneficial metabolites, (3) inhibiting pathogen colonization and proliferation, and (4) improving immunotherapy efficacy [224]. Postbiotics released by *Lacticaseibacillus paracasei* can enhance cancer cell sensitivity to ICI therapy, thereby working synergistically with immunotherapy to inhibit tumor growth. Specifically, the microbial metabolite phytosphingosine can induce activation of innate immune signaling pathways (including upstream MYD88/NF‐κB and downstream NLRC5), thereby promoting T cell‐mediated antitumor immune responses and ultimately inhibiting tumor growth [225].

#### 5.2.3 Antibiotics Associated With Poor Efficacy of Immunotherapy

Multiple studies have demonstrated that ATBs are significantly associated with diminished efficacy, worse prognosis, and increased risk of adverse events in cancer patients receiving immunotherapy. In particular, the use of broad‐spectrum ATBs is significantly associated with resistance to anti‐PD‐1/PD‐L1 therapy, adverse reactions, and reduced PFS and OS [198, 218, 226]. Research has demonstrated that cancer patients receiving ATBs concurrently with anti‐PD‐1/PD‐L1 therapy exhibited significantly worse prognosis compared to those receiving anti‐PD‐1/PD‐L1 therapy alone, while supplementation with *AKK* could effectively restore the efficacy of immunotherapy [116]. A clinical study revealed that the use of ATBs, especially fluoroquinolones, in muscle‐invasive bladder cancer patients receiving neoadjuvant Pembrolizumab therapy was significantly associated with lower complete response rates and higher recurrence rates, highlighting the significant negative impact of ATB treatment on immunotherapy efficacy [227]. Notably, prior ATB (pATB) administration also adversely affects immunotherapy efficacy. Related studies have demonstrated that in advanced GC patients receiving PD‐1 inhibitor therapy, pATB was associated with reduced gut microbial diversity, suppression of *Lactobacillus gasseri*, and dysregulation of circulating exhausted CD8^+^ T cells after treatment, which subsequently resulted in significantly decreased PFS and OS. These findings indicate that clinicians should thoroughly evaluate whether advanced GC patients are planning to receive PD‐1 inhibitor therapy prior to prescribing ATB [228]. Research by Eng et al. corroborated these findings, indicating that pATB use (especially fluoroquinolones) was associated with significantly worse OS in elderly cancer patients receiving ICI therapy [229]. Additionally, a retrospective cohort study investigating the role of ATBs in HCC patients receiving ICIs demonstrated that the concurrent use of ATBs and ICIs was significantly associated with higher mortality in advanced HCC patients [230]. Finally, in LC patients receiving anti‐PD‐1/PD‐L1 therapy, ATB use led to decreased gut microbial diversity, which was significantly associated with an increased risk of irAEs [231].

However, another clinical study has indicated that compared to CD19‐targeted CAR‐T cell therapy alone, patients receiving vancomycin pretreatment before CAR‐T cell therapy achieved significantly enhanced tumor control effects, a phenomenon possibly related to increased cross‐presentation of tumor‐associated antigens, suggesting that vancomycin‐modulated gut microbiome composition may constitute one of the key factors influencing the efficacy of CAR‐T cell immunotherapy [232]. This finding contrasts with the aforementioned association between ATBs and poor immunotherapy efficacy, likely because vancomycin exerts distinct mechanisms of action compared to other broad‐spectrum ATBs. Vancomycin exhibits selective activity against gram‐positive and anaerobic bacteria while remaining ineffective against gram‐negative bacteria. Previous studies have demonstrated that ATBs with antianaerobic activity are associated with lower cancer‐related mortality risk compared to those with antiaerobic activity [230]. A separate study demonstrated that modulating the gut microbiome with vancomycin may enhance the efficacy of CAR‐T cell therapy [232]. The vancomycin‐modulated microbiome exhibited decreased α‐diversity, reduced abundance of *Ruminococcaceae* and *Lachnospiraceae*, decreased SCFA concentrations, and expansion of bacterial strains such as *AKK*. Notably, reduced SCFAs can impair antigen presentation, whereas *AKK* has been confirmed to influence immunotherapy responses [116]. Furthermore, vancomycin treatment has been shown to stimulate the expansion of AH1‐specific CD8^+^ T cells and promote significant increases in CAR‐T cells, thereby exerting a protective effect on CAR‐T cell therapy efficacy. In conclusion, while ATBs generally exhibit detrimental effects on immunotherapy efficacy, notable exceptions do exist.

#### 5.2.4 Other Microbial Manipulation Strategies Affecting Cancer Immunotherapy

Beyond the aforementioned strategies, targeted therapeutic approaches against specific microbes and engineered microorganisms also significantly contribute to enhancing the efficacy of immunotherapy in tumor tissues. Research suggests that interventions targeting *F. nucleatum* in combination with immunotherapy and autophagy inhibitors hold promise as an effective treatment strategy for breast cancer [210]. In summary, various microbial intervention strategies can significantly enhance or inhibit the efficacy and adverse reactions of cancer immunotherapy, suggesting that rational combinations of microbiome‐targeted therapies and immunotherapy may provide novel therapeutic approaches for anticancer treatment. Anti‐PD‐L1 therapy plays a crucial role in cancer immunotherapy, yet its efficacy is often diminished by the immunosuppressive effects mediated by TDEs. To address this issue, researchers have developed an engineered probiotic that specifically targets TDEs and effectively activates antitumor immune responses, providing a novel‐targeted immunotherapy strategy for clinical applications [211]. Mao et al. [233] developed an engineered *Lactobacillus acidophilus* (LH@LA) strain capable of inducing the polarization of M2‐type macrophages (anti‐inflammatory) into M1‐type macrophages (proinflammatory) [234], thereby enhancing antitumor immune responses. This engineered bacterium, equipped with a specialized protective coating, can effectively ameliorate the local immunosuppressive microenvironment by metabolizing L‐lactic acid in the TME.

### 5.3 Microbiome Research and Personalized Cancer Immunotherapy

Microorganisms, as promising therapeutic carriers, are being strategically engineered for personalized cancer immunotherapy with the aim of enhancing their clinical efficacy. Based on systematic research evidence, personalized cancer vaccines and TCR‐T cell adoptive transfer therapy have demonstrated considerable promise as potential therapeutic strategies for personalized immunotherapy in cancer patients [235]. Multiple studies across different tumor types, including esophageal cancer, melanoma, NSCLC, and bladder cancer, have demonstrated that patients receiving specific cancer vaccines or neoantigen‐specific T cell therapy exhibited significantly improved ORR, enhanced immunotherapy responses, prolonged survival duration, and reduced risks of tumor metastasis and recurrence [235]. Similar outcomes were observed in RCC studies, where researchers employed personalized cancer vaccines as a postoperative adjuvant treatment strategy. The results demonstrated that these vaccines could induce durable antigen‐specific T‐cell immune responses in RCC patients and significantly reduce the risk of tumor recurrence [236]. Despite the encouraging clinical potential of personalized tumor immunotherapy strategies, their translation into clinical applications still faces numerous challenges, including limited diversity of tumor‐specific antigens, constraints in adjuvant selection, inefficient antigen presentation, and interference from the immunosuppressive TME on vaccine efficacy [212]. In response to these challenges, researchers are actively exploring cancer vaccines based on bacterial or viral vectors. In these studies, bacteria and viruses or their derivatives are precisely engineered and utilized as vaccine delivery systems. This biohybrid approach fully leverages multiple unique advantages of microorganisms, including genomic editability, endogenous adjuvant properties, potent activation of the innate immune system, and the ability to modulate the tumor immune microenvironment, thereby significantly enhancing the therapeutic effects of personalized cancer vaccines [212, 237]. Specifically, research indicates that demi‐bacteria from *Bacillus*, after specialized processing, can serve as efficient carriers for delivering cancer vaccines. In vivo experiments have confirmed that mouse models vaccinated with demi‐bacteria‐based biomimetic vaccines exhibited synergistic activation of cellular and humoral immune responses, thereby significantly enhancing the potency of antitumor immune responses [238]. In conclusion, the integration of microbiome technology with personalized tumor immunotherapy strategies holds promise for producing synergistic antitumor effects that are significantly superior to traditional monotherapies, thus offering new research directions and clinical translation potential for precision cancer treatment.

### 5.4 Challenges and Opportunities of Microbiome Research in Cancer Immunotherapy

Microbiome research has demonstrated immense potential in enhancing the efficacy of cancer immunotherapy and reducing adverse effects, yet numerous challenges remain, making further optimization of combined treatment strategies involving microbiome modulation and immunotherapy a promising approach for anticancer therapy. While extensive research has explored the associations between the microbiome and cancer treatment across various malignancies, the molecular mechanisms underlying these associations remain inadequately elucidated in many cancer types [239], making it crucial to thoroughly investigate the potential mechanisms by which the microbiome influences immunotherapy. Since the composition and stability of the gut microbiome are susceptible to various factors, including dietary patterns, ATB use, and intestinal health status, rigorous assessment and effective control of potential confounding factors are necessary when utilizing it as a biomarker for immunotherapy response [240]. Meanwhile, different microbial communities may exert substantially different effects in various physiological and pathological environments: commensal bacteria in the gastrointestinal tract can promote immune system development and maintain immune homeostasis, whereas in the lung TME, they may participate in shaping inflammatory responses and accelerating tumor proliferation [70, 241]. Therefore, comprehensive analysis of the functional characteristics and regulatory mechanisms of different bacterial communities is of critical significance for the precise development of microbiome‐mediated antitumor immunotherapy strategies [241]. Furthermore, the aforementioned microbial vector‐mediated cancer vaccine delivery systems still face numerous technical bottlenecks, including a lack of standardized production processes and rigorous quality control systems, the need to optimize combination therapy regimens to expand clinical applications, and insufficient systematic safety evaluations and clinical translation studies [212]. For instance, bacterial‐derived outer membrane vesicles as potential vaccine delivery platforms still face technical barriers such as insufficient antigen loading capacity, susceptibility to host antibody‐mediated clearance, and high endotoxin content, necessitating structural modifications and preparation process innovations to enhance the clinical translation prospects of outer membrane vesicles [237]. Despite the numerous challenges facing the clinical application of the microbiome in cancer immunotherapy, in‐depth exploration of microbe–host interaction mechanisms and systematic resolution of the aforementioned technical issues will establish new avenues for the clinical translation of microbiome‐based therapies.

In conclusion, microbiome research provides novel biomarkers and intervention strategies for tumor immunotherapy, demonstrating broad clinical application potential. However, current research still faces challenges such as unclear mechanisms, insufficient standardization, and substantial individual variations. Future efforts should focus on multiomics integration, clinical trial optimization, and advances in engineered bacterial technologies to drive microbiome research toward precise and personalized tumor immunotherapy.

## 6 Future Perspective and Conclusions

**FIGURE 5:**
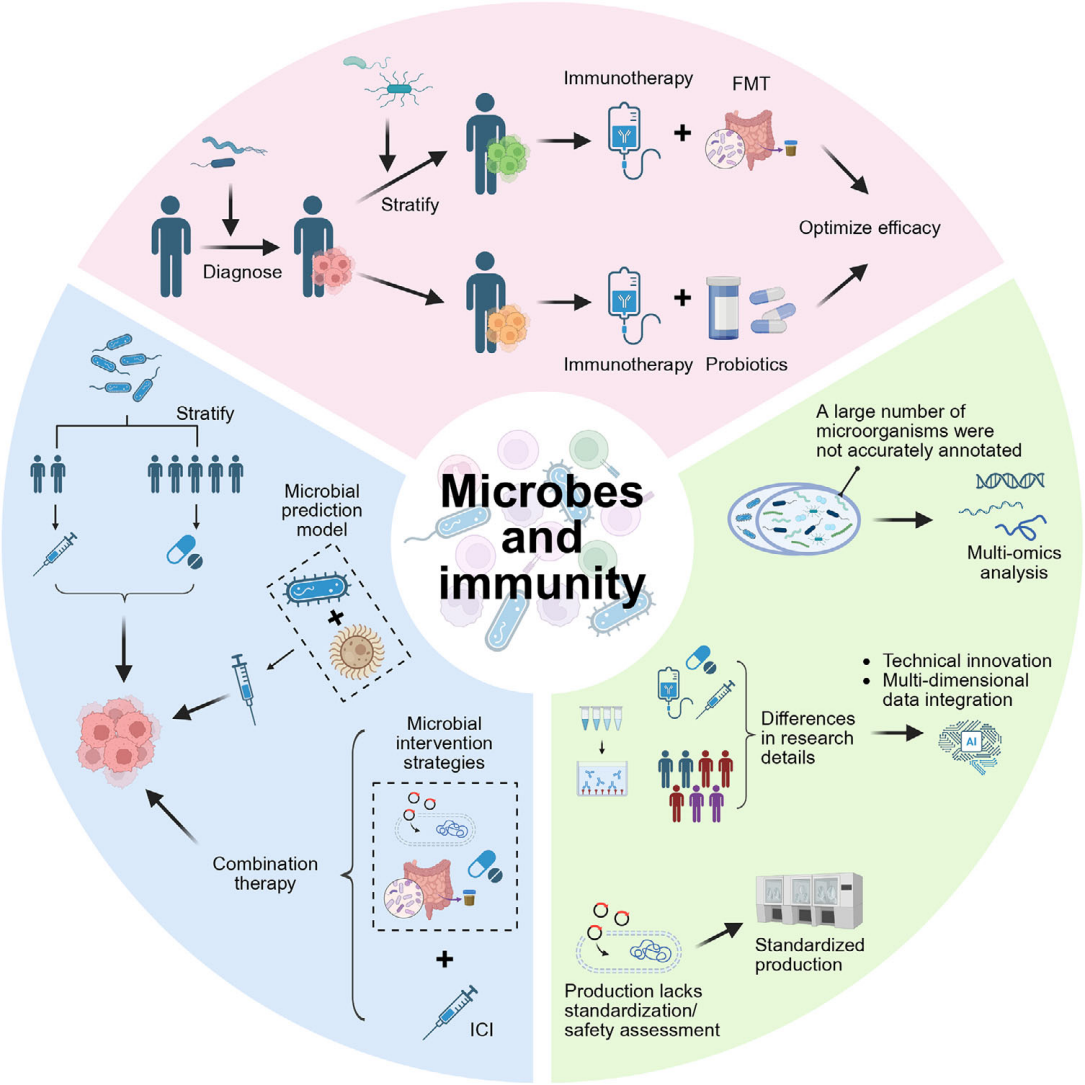
Exploratory directions for microbiome research to further advance cancer immunotherapy. Microbiome research holds significant importance for cancer diagnosis, guiding personalized treatment regimens for cancer patients, and predicting the efficacy and prognosis of cancer immunotherapy with greater precision. Combined treatment approaches involving microbiome interventions and immunotherapy offer promising and innovative therapeutic strategies for cancer treatment. However, current research faces numerous methodological and technical limitations that require systematic exploration and rigorous improvements in research protocols. This figure was created using the tools provided by Biorender.com (accessed on 08/04/2025). FMT, fecal microbiome transplantation; ICI, immune checkpoint inhibitor.

Microbiome research has emerged as a frontier area in cancer immunotherapy, demonstrating profound impacts on treatment efficacy through its regulation of the tumor immune microenvironment. Current studies have definitively revealed significant associations between the microbiome and immunotherapy responses, providing new theoretical foundations and potential intervention tools for optimizing clinical treatment strategies. However, this field still faces numerous challenges, including insufficient elucidation of molecular mechanisms and the absence of standardized evaluation methods for intervention strategies. Therefore, future research should focus on in‐depth exploration of microbiome‐related biomarkers, optimization of combination therapies, and development of precise individualized intervention protocols. With the continued advancement of multiomics technology integration and large‐scale prospective clinical trials, microbiome research will further drive cancer immunotherapy toward precision medicine, providing solid scientific evidence for developing patient‐specific personalized treatment plans, improving therapeutic efficacy, and enhancing long‐term prognosis (Figure 5).

### 6.1 Significance of Microbiome Research for Cancer Immunotherapy

Microbiome research has opened new avenues for cancer immunotherapy by elucidating the molecular mechanisms of host–microbe interactions in immune system regulation, thereby systematically advancing the development of treatment strategies toward combination, individualization, and precision approaches. Numerous studies have confirmed that the microbiome constitutes a critical foundation for the development of novel predictive tools. Existing evidence indicates that gut bacteria significantly influence ICI treatment responses, while a recent study demonstrated that prediction models based on fungal markers exhibit markedly superior accuracy compared to traditional bacterial markers in cross‐regional cohorts, suggesting that integrating bacterial and fungal markers can yield more precise prediction systems [242]. Additionally, the gut microbiome not only regulates the efficacy of ICIs through multiple mechanisms but also demonstrates considerable potential as an important target for combined cancer immunotherapy. Engineered bacteria developed through synthetic biology technologies have demonstrated effective remodeling of the TME in preclinical studies, thereby enhancing the antitumor activity of immune cells [145]; simultaneously, FMT, which transfers microbiome from “good responders” to nonresponders, has been proven to significantly enhance response rates to ICI therapy [65, 243]. The widespread use of ATBs significantly disrupts the ecological balance of the gut microbiome, consequently leading to markedly decreased efficacy of CAR‐T cell therapy, indicating that microbial intervention strategies require careful consideration of their dual role in combined immunotherapy regimens in clinical applications [244]. Meanwhile, specific ecological characteristics of the microbiome can serve as novel biomarkers, effectively guiding the assessment of patient treatment response stratification and the development of individualized and precision treatment plans. For instance, the species‐level gut microbiome prediction (SPEED) model, based on key bacterial taxa including *Lachnospiraceae* bacterium and *Bacteroides intestinalis*, can accurately predict clinical responses of CRC patients to neoadjuvant chemoradiotherapy combined with immunotherapy, attaining a predictive performance of up to 98.8% in validation cohorts [245]. Microbiome research demonstrates significant potential in evaluating treatment responses, identifying resistance mechanisms, and discovering synergistic therapeutic targets, advancements that are expected to facilitate the development of microbiome‐based innovative anticancer drugs [246]. In summary, microbiome research is providing multidimensional innovative approaches and implementation strategies for individualized and precision cancer treatment.

### 6.2 Current Research Limitations and Future Directions

Despite the growing attention to research on microbiome and immunotherapy relationships, current studies still face multiple limitations. First, current microbiome research technologies remain significantly constrained. Existing multiomics technologies (such as metagenomics) still rely on limited reference genome databases for microbial functional analysis, resulting in numerous microbial sequences that cannot be accurately annotated, thereby impeding comprehensive analyses of deep associations between microbiome functions and immune regulatory mechanisms [247]. Second, due to limited clinical sample sizes and insufficient long‐term follow‐up data, most studies on the relationship between the microbiome and immunotherapy have not yet reached a fully mature stage. Additionally, heterogeneity in detection methods, diverse confounding factors in treatment regimens, and physiological differences between individual patients collectively contribute to substantial heterogeneity and compromised comparability among different research findings [240]. Furthermore, due to the lack of standardized protocols and long‐term safety evaluation systems for microbial intervention strategies (such as engineered bacteria and OVs), the clinical translation of these novel therapeutic approaches encounters significant barriers [212]. To address these challenges, future research directions should emphasize technological innovation and multidimensional data integration, aiming to achieve highly correlated and precise predictions of microbiome functions and immunotherapy responses through technological iterations and in‐depth interdisciplinary collaboration [248, 249]. With the expanded application of emerging technologies such as metabolomics, metagenomics, and single‐cell sequencing, researchers will be better equipped to explore the molecular mechanisms underlying microbiome and cancer immunotherapy associations, thereby establishing solid theoretical foundations for developing combination treatment strategies. Finally, there is an urgent need to optimize standardized production and quality control processes for engineered microorganisms and to advance large‐scale multicenter clinical trials to accelerate clinical translation, thereby facilitating the development of individualized microbial intervention strategies and microbiome‐based precision cancer immunotherapy protocols, ultimately offering cancer patients safer, more effective, and individualized comprehensive treatment strategies.

### 6.3 Development Prospects of Microbiome and Cancer Immunotherapy Research

Microbiome research has infused new vitality into the field of cancer immunotherapy, offering a series of innovative strategies to enhance treatment precision and overcome therapeutic resistance. In the future, development in this field will focus on the development of predictive biomarker tools, the optimization of combination treatment strategies and individualized precision intervention protocols, and the development of novel therapeutic agents, thereby promoting more precise and effective cancer immunotherapy while reducing patients’ disease and treatment burdens. First, microbiome prediction models have demonstrated encouraging predictive performance [242, 245], enabling the precise stratification of cancer patients for personalized treatment planning. These findings have laid a solid foundation for the development of efficient and reliable noninvasive prognostic assessment tools. Meanwhile, bacterial therapies significantly enhance the efficacy of ICIs through precisely targeted regulation of the TME. For example, oncolytic mineralizing bacteria activate innate immunity through multiple signaling pathways, thereby promoting immune cell infiltration and inducing abscopal antitumor effects, thus demonstrating significant therapeutic potential [250]. Microbiome‐based combination therapies hold promise for overcoming the efficacy limitations of conventional treatments, offering new therapeutic options for patients with refractory tumors and demonstrating promising development prospects. With the widespread application of artificial intelligence and machine learning technologies, researchers can discover novel biomarkers and therapeutic targets from vast and complex microbiome data with unprecedented speed and precision [251], thereby establishing scientific foundations for optimizing clinical trial designs and developing new clinical therapeutics. As microbiomics becomes more deeply integrated with immunotherapy, “microbiome–immune” combination therapy is poised to become the next milestone in cancer precision medicine, providing innovative pathways to improve immunotherapy response rates and expand the population of patients who benefit, offering bright prospects for future development.

In conclusion, this review comprehensively elucidates the complex interaction networks and potential molecular mechanisms between the microbiome across different human tissue sites and cancer immunotherapy. This article emphasizes the bidirectional regulatory effects between the gut microbiome and the tumor immune microenvironment, and thoroughly analyzes the molecular mechanisms by which gut microbes influence cancer immunotherapy efficacy through multiple signaling pathways, thereby providing a theoretical foundation for developing precision cancer treatment strategies targeting the gut microbiome. Additionally, this review focuses on the emerging research field of the intratumoral microbiome, where multiple studies have confirmed its close association with local immune responses across various cancer types and its significant regulatory role in immunotherapy responses within the TME, demonstrating potential clinical value as prognostic biomarkers for cancer immunotherapy. This article systematically discusses the microbiome as a frontier research direction in cancer therapy, highlighting the unique advantages of its precise regulatory strategies in enhancing immunotherapy efficacy. Based on existing evidence, future research should prioritize the comprehensive elucidation of molecular mechanisms underlying microbiome–immune interactions, with particular emphasis on the roles of microbial metabolites and their signaling pathways in immune activation and suppression. Concurrently, efforts should focus on advancing the clinical translation of microbiome‐based predictive biomarker, and establishing multidimensional, pan‐cancer microbial scoring systems to guide personalized immunotherapy applications. Additionally, research priorities should include optimizing microbial intervention strategies, including the development of NGPs, engineered bacteria, and standardized FMT protocols, while effectively integrating these approaches with existing therapies such as ICIs and CAR‐T cell therapy. Conducting large‐scale prospective clinical trials, integrating multiomics technologies with artificial intelligence approaches, and validating the clinical benefits and feasibility of microbiome interventions represent both cost‐effective and essential steps toward achieving the clinical translation of microbial therapeutics. This review objectively evaluates the technical bottlenecks and future development challenges facing current microbiome research, clarifies the enormous application potential of microbiome‐mediated antitumor immune response mechanisms in precision cancer therapy, and establishes a theoretical framework for integrating innovative treatment strategies targeting the microbiome into contemporary cancer immunotherapy approaches.

## Author Contributions

Writing‐original draft, Anqi Lin and Minying Xiong; Conceptualization, Bufu Tang, Pengpeng Zhang, and Peng Luo; Investigation, Anqi Lin, Minying Xiong, and Aimin Jiang; Writing‐review and editing, Anqi Lin, Minying Xiong, Aimin Jiang, Li Chen, Lihaoyun Huang, Kailai Li, Hank Z. H. Wong, Jian Zhang, Zaoqu Liu, Quan Cheng, Bufu Tang, Pengpeng Zhang, and Peng Luo; Visualization, Anqi Lin and Minying Xiong. All the authors have read and agreed to the published version of the manuscript.

## Ethics Statement

The authors have nothing to report.

## Conflicts of Interest

The authors declare no conflicts of interest.

## Data Availability

The authors have nothing to report.
